# Data on molecular docking of tautomers and enantiomers of ATTAF-1 and ATTAF-2 selectivty to the human/fungal lanosterol-14α-demethylase

**DOI:** 10.1016/j.dib.2020.105942

**Published:** 2020-06-27

**Authors:** Hamid Irannejad, Saeed Emami, Hassan Mirzaei, Seyedeh Mahdieh Hashemi

**Affiliations:** a Department of Medicinal Chemistry, Faculty of Pharmacy, Mazandaran University of Medical Sciences, Sari, Iran; b Pharmaceutical Sciences Research Center, Mazandaran University of Medical Sciences, Sari, Iran; c Ischemic Disorders Research Center, Golestan University of Medical Sciences, Gorgan, Iran

**Keywords:** Molecular docking, Lanosterol-14α-demethylase, Selectivity, Antifungal

## Abstract

The data have been obtained for tautomers and enantiomers of ATTAF-1 and ATTAF-2 that were developed based on antifungal standard drugs with triazole scaffold. These compounds were docked into the human and fungal lanosterol-14α-demethylase. In order to validate the data, 8 standard triazole antifungal drugs (Fluconazole, Itraconazole, Posaconazole, Ravuconazole, Albaconazole, Voriconazole, Isavuconazole and Efinaconazole) were also docked into the human and fungal lanosterol-14α-demethylase. The binding conformations of these molecules and their interactions with lanosterol-14α-demethylase may inform the development of further small molecule lanosterol-14α-demethylase inhibitors with significant selectivity toward this enzyme. The analysis has done on the basis of type of interactions (bond type and distance). The length of the Fe-N coordination bond for (*R*)-N2-ATTAF-1 and (*S*)-N1-ATTAF-2 complexes is obtained 6.36 and 4.19 Å, respectively and about 2 Å in the other tautomer and enantiomer complexes, reflecting the lower basicity of the N-4 atom in the 1,2,4-triazole ring of (*R*)-N2-ATTAF-1 and (*S*)-N1-ATTAF-2 in comparison with the N-4 atom in the 1,2,4-triazole ring in other tautomers and enantiomers and supporting higher selectivity of (*R*)-N2-ATTAF-1 and (*S*)-N1-ATTAF-2 towards the target CYP51 enzymes vs. human. Interestingly, we have investigated unfavorable interactions (donor-donor) with TRP239 and MET378 for (*R*)-N2-ATTAF-1 and (*S*)-N1-ATTAF-2, respectively. These unfavorable interactions also have been seen in case of posaconazole and isavuconazole. The data presented in this article are related to the research paper entitled "*In silico* prediction of ATTAF-1 and ATTAF-2 selectivity towards human/fungal lanosterol 14α-demethylase using molecular dynamic simulation and docking approaches".

Specifications table

**Table:** 

Subject	Computational and *in silico* Chemistry
Specific subject area	Docking studies
Type of data	Table Figure
How data were acquired	ChemDraw Ultra 8.0 and Molecular docking (AutoDockTools-1. 5. 4)
Data format	Raw Analyzed
Parameters for data collection	The docking of the tautomeric and enantiomeric forms of ATTAF-1 and ATTAF-2 was targeted at a 6 Å radius area that encompassed the heme of lanosterol-14α-demethylase (PDBs: 5JLC, 5V5Z, 3LD6) using bond type and distance and interactions of proteins with the ligands.
Description of data collection	The Proteins were collected from Protein Data Bank (www.rcsb.org). Ligands 3D structures were obtained by using ChemDraw Ultra 8.0 and energy minimized using PM3 force field. The docking was done using AutoDockTools-1. 5. 4. Searching algorithm in docking study was lamarckian genetic algorithm.
Data source location	Institution: Mazandaran University of Medical Sciences City/Town/Region: Sari, Farahabad Country: Iran
Data accessibility	Data is with this article.
Related research article	H. Irannejad, S. Emami, H. Mirzaei, S. M. Hashemi, *In silico* prediction of ATTAF-1 and ATTAF-2 selectivity towards human/fungal lanosterol 14α-demethylase using molecular dynamic simulation and docking approaches, https://doi.org/10.1016/j.imu.2020.100366.

## Value of the data

• The lanosterol 14α-demethylase has been identified as a molecular target for the treatment of fungal diseases.
• The modeling data was produced to rationalize the structural necessities for lanosterol 14α-demethylase inhibition.
• The binding conformations of the ATTAF-1 and ATTAF-2, their interactions with human and fungal lanosterol 14α-demethylase and coordination bond distance may inform further studies focused on the development of lanosterol 14α-demethylase inhibitors.
• Novel synthetic analogues with improved bioactivity and minimized side effects can be expanded against this target by using this *in silico* docking data and research time can be minimized significantly.
• This dataset can be useful to model other potent antifungal agents in future and researchers in pharmaceutical chemistry can gain from the data.

## 1 Data description

**Fig. 1:**
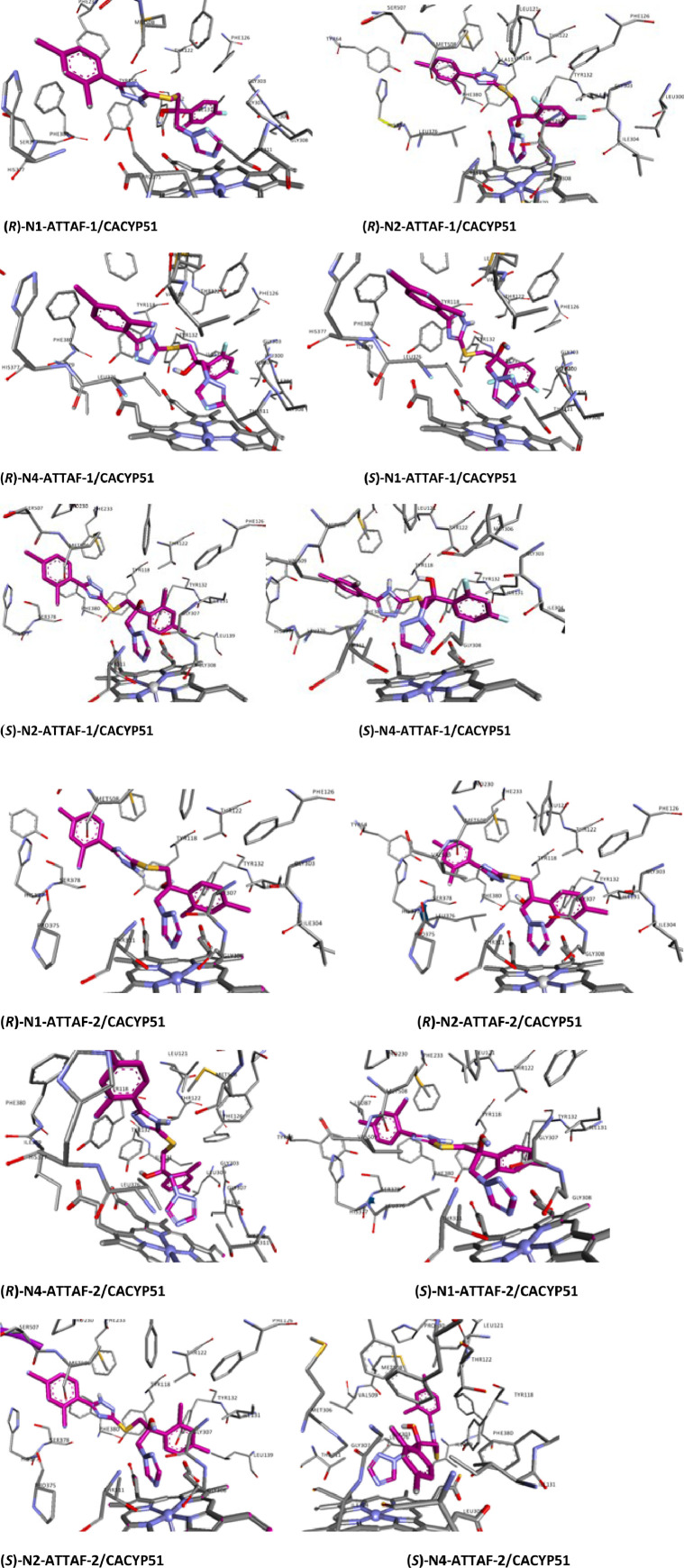
Ligand interaction map of the predicted binding mode of tautomers and enantiomers of ATTAF-1 and ATTAF-2 in the active site *Candida albicans* CYP51.

**Fig. 2:**
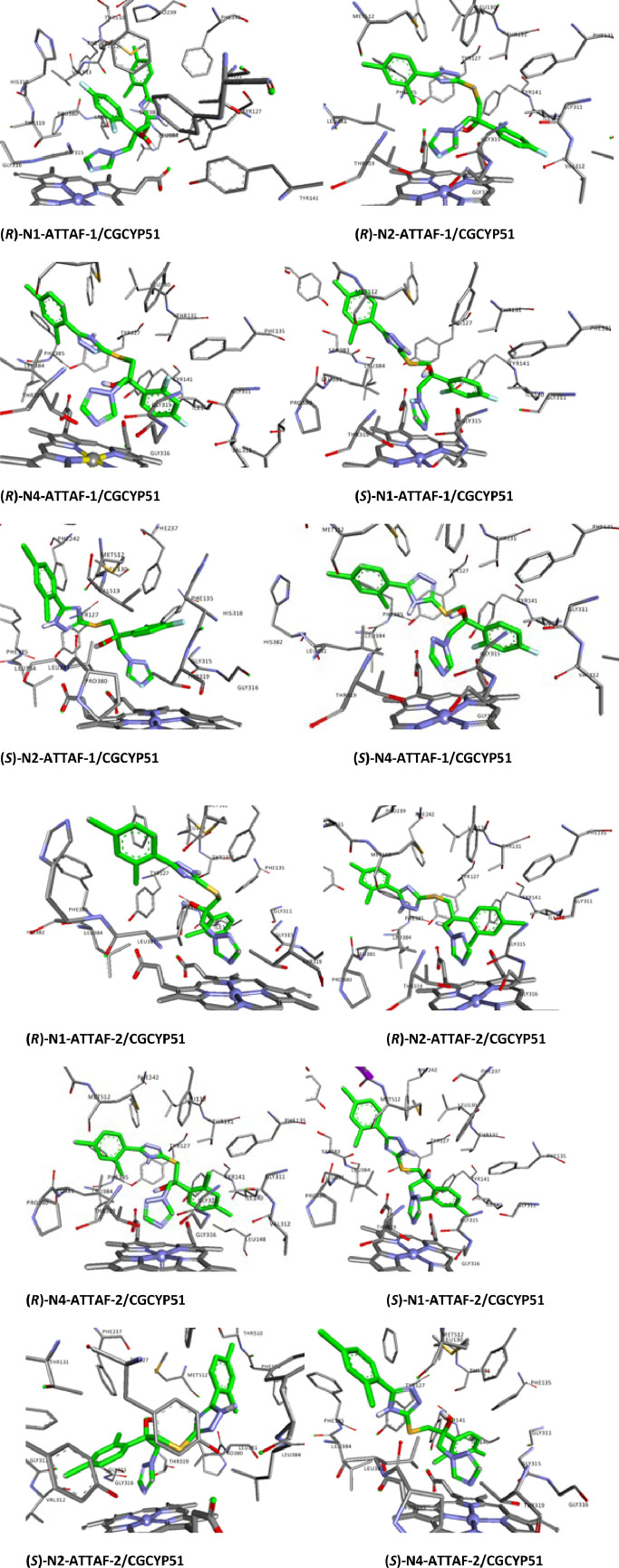
Ligand interaction map of the predicted binding mode of tautomers and enantiomers of ATTAF-1 and ATTAF-2 in the active site *Candida glabrata* CYP51.

**Fig. 3:**
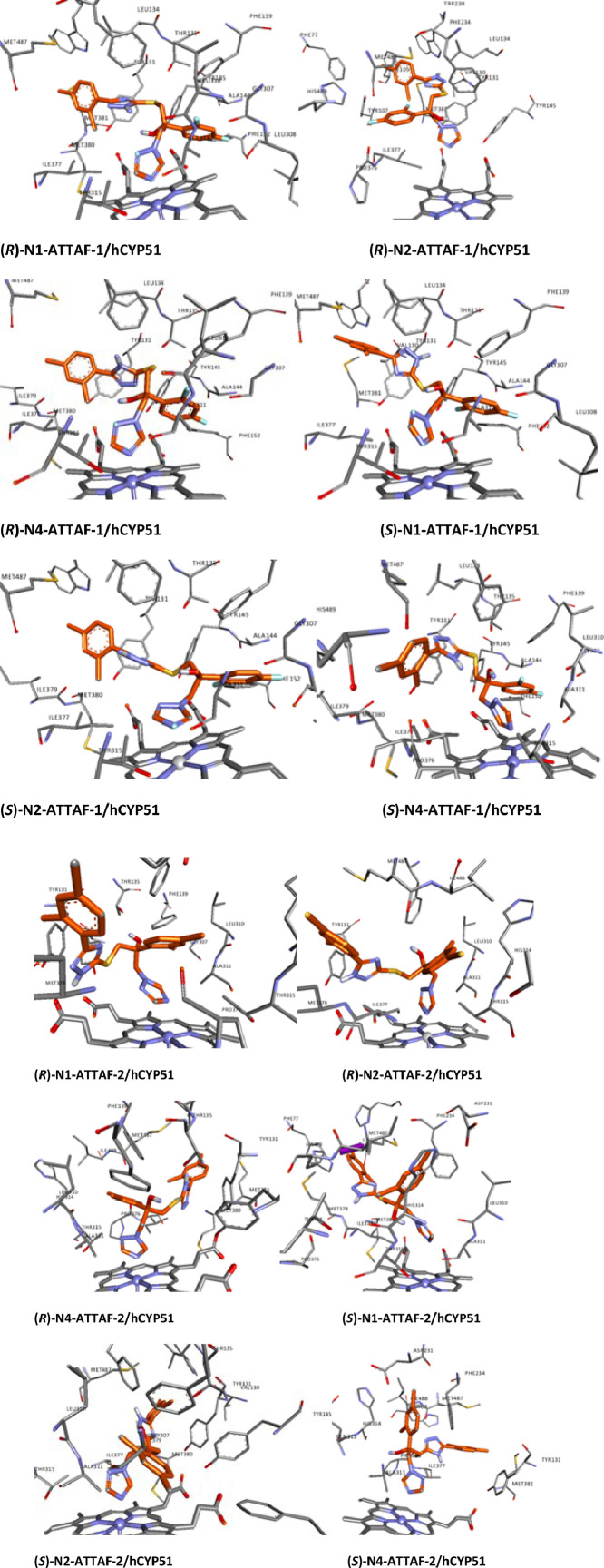
Ligand interaction map of the predicted binding mode of tautomers and enantiomers of ATTAF-1 and ATTAF-2 in the active site human CYP51.

Lanosterol-14α-demethylase (CYP51) is found in mycobacteria, fungi, plants, animals and humans. This enzyme is required for biosynthesis of sterol in eukaryotes and is the major target for azole antifungal agents [1,2]. In mammals, lanosterol-14α-demethylase is the enzyme that catalyzes lanosterol to cholesterol conversion, which is necessary to maintain a variety of metabolic functions [3]. An ideal antifungal agent should have minimal effect on human CYP51 enzymes while keeping potent inhibition of fungal enzyme to reduce the side effects [4]. Lanosterol-14α-demethylase consists of an iron protoporphyrin unit in its active site. At the molecular level, N-4 in the 1,2,4-triazole ring selectively coordinates to the lanosterol-14α-demethylase heme iron and cause the prevention of the fungal ergosterol biosynthesis pathway [5]. In order support a medicinal chemistry campaign to develop potent azole antifungal agents with high CYP51 affinity, we have previously synthesized and reported a series of novel fluconazole analogues, with the most promising ones introduced as ATTAF-1 and ATTAF-2 [6] and provided the computational-based docking and MD simulation outputs for all tautomeric and enantiomeric forms of ATTAF-1 and ATTAF-2 plus 8 antifungal standard drugs were docked into the human and fungal lanosterol-14α-demethylase [9]. Here, our studies provide important protein-ligand interaction information for the further development of lanosterol-14α-demethylase inhibitors. In this article Table 1 provides the details about the targets and their description. Table 2 gives the coordinates of the cubic box used to dock ATTAF-1 and ATTAF-2 to the fungal and human CYP51. Table 3 gives the length of the Fe-N coordination bond. Table 4 provides tautomers and enantiomers of ATTAF-1 and ATTAF-2 interactions with fungal and human CYP51. To point to the N-4 coordination with heme iron, 3D interactions of all tautomeric and enantiomeric forms of ATTAF-1 and ATTAF-2 with the target enzymes are shown in the Fig. 1, Fig. 2, Fig. 3. 2D interactions of all tautomeric and enantiomeric forms of ATTAF-1, ATTAF-2 and 8 standard triazole antifungal drugs with the target enzymes are shown in the supplemental file .

**Table 1:** List of targets.

Entry	PDB ID	Resolution (Å)	Description	RMSD (Å)
1	5V5Z	2.9	Structure of CYP51 from the pathogen *Candida albicans*[7]	1.71
2	5JLC	2.4	Structure of CYP51 from the pathogen *Candida glabrata*[7]	1.58
3	3LD6	2.8	Crystal structure of human lanosterol 14alpha-demethylase (CYP51) in complex with ketoconazole [8]	1.04

**Table 2:** Coordinates of the cubic box used to dock ATTAF-1 and ATTAF-2 to the fungal and human CYP51.

Entry	Side	Coordinate		
		CACYP51	CGCYP51	hCYP51
1	X	−42.490	−40.056	42.287
2	Y	−13.523	74.850	4.969
3	Z	26.334	−23.564	1.219

**Table 3:** Length of the Fe-N coordination bond.

Entry	Compound	Coordination Bond Distance (Å)		
		CACYP51	CGCYP51	hCYP51
1	(*R*)-N1-ATTAF-1	2.52	2.93	2.17
2	(*R*)-N2-ATTAF-1	2.21	2.63	**6.36**
3	(*R*)-N4-ATTAF-1	2.91	2.25	1.92
4	(*S*)-N1-ATTAF-1	2.56	2.44	2.83
5	(*S*)-N2-ATTAF-1	2.29	2.89	2.92
6	(*S*)-N4-ATTAF-1	2.53	2.80	2.70
7	(*R*)-N1-ATTAF-2	2.32	2.83	2.67
8	(*R*)-N2-ATTAF-2	2.12	2.85	2.19
9	(*R*)-N4-ATTAF-2	2.20	2.26	2.03
10	(*S*)-N1-ATTAF-2	2.33	2.80	**4.19**
11	(*S*)-N2-ATTAF-2	2.45	2.86	2.04
12	(*S*)-N4-ATTAF-2	2.74	2.74	3.12

**Table 4:** Tautomers and enantiomers of ATTAF-1 and ATTAF-2 interactions with fungal and human CYP51.

Entry	Target	Ligand	Interaction	Type of interaction	Bond distance (Å)
1.	**5V5Z**	(*R*)-N1-ATTAF-1	Tyr132 Phe233 Met508 Tyr118 Phe233	H-Bond Pi-Pi stacking Pi-Sulfur Pi-Sulfur Pi-Sulfur	2.34 5.13 5.85 3.60 5.96
		(*R*)-N2-ATTAF-1	Tyr132 Phe380 Met508 Phe228	H-Bond Pi-Pi stacking Pi-Sulfur Pi-Sulfur	2.32 4.92 5.45 5.87
		(*R*)-N4-ATTAF-1	Phe380 Phe233 Tyr118 Ser378	Pi-Pi stacking Pi-Pi stacking Pi-Pi stacking Pi-Pi stacking	4.93 5.81 3.44 4.17
		(*S*)-N1-ATTAF-1	Tyr132 Tyr118 Phe380	H-Bond Pi-Pi stacking Pi-Pi stacking	3.16 3.54 5.08
		(*S*)-N2-ATTAF-1	Tyr132 Tyr118 Phe380	H-Bond Pi-Pi stacking Pi-Pi stacking	3.21 3.71 5.91
		(*S*)-N4-ATTAF-1	Ser378 Phe380 Phe233 Tyr118	Pi-Pi stacking Pi-Pi stacking Pi-Pi stacking Pi-Pi stacking	4.14 4.96 5.86 3.52
		(*R*)-N1-ATTAF-2	Phe233 Phe228	Pi-Pi stacking Pi-Sulfur	5.75 5.48
		(*R*)-N2-ATTAF-2	Tyr118 Phe380	Pi-Pi stacking Pi-Pi stacking	5.84 5.31
		(*R*)-N4-ATTAF-2	Tyr132 Tyr118 Phe228 Met508	H-Bond Pi-Pi stacking Pi-Sulfur Pi-Sulfur	2.59 4.45 5.50 5.41
		(*S*)-N1-ATTAF-2	Phe233 Tyr118 Met508	Pi-Pi stacking Pi-Sulfur Pi-Sulfur	5.01 4.35 5.62
		(*S*)-N2-ATTAF-2	Tyr118 Ser378	H-Bond H-Bond	2.64 2.10
		(*S*)-N4-ATTAF-2	Tyr132 Phe380 Tyr118	H-Bond Pi-Pi stacking Pi-Pi stacking	2.94 5.93 3.72
2.	**5JLC**	(*R*)-N1-ATTAF-1	Tyr127 Phe237	H-Bond Pi-Pi stacking	1.99 5.01
		(*R*)-N2-ATTAF-1	Tyr127 Phe237 Phe135	Pi-Pi stacking Pi-Sulfur Pi-Sulfur	5.89 5.02 5.12
		(*R*)-N4-ATTAF-1	Phe242 Tyr127 Phe135 Tyr141 Met512	Pi-Pi stacking Pi-Pi stacking Pi-Pi stacking Pi-Sulfur Pi-Sulfur	5.28 5.41 5.44 5.39 4.82
		(*S*)-N1-ATTAF-1	Tyr141	H-Bond	2.61
		(*S*)-N2-ATTAF-1	His318 Thr319 Ser383 Phe242 Met512 Tyr127	H-Bond H-Bond H-Bond Pi-Pi stacking Pi-Sulfur Pi-Sulfur	2.68 3.58 1.98 5.31 5.29 4.20
		(*S*)-N4-ATTAF-1	Tyr141 Phe242 Tyr127 Tyr141 Tyr127 Met512	2 H-Bond Pi-Pi stacking Pi-Pi stacking Pi-Sulfur Pi-Sulfur Pi-Sulfur	2.60, 3.58 5.32 4.04 4.86 3.93 4.79
		(*R*)-N1-ATTAF-2	Tyr141	Pi-Pi stacking	5.42
		(*R*)-N2-ATTAF-2	Ser383 Phe385 Met512	H-Bond Pi-Pi stacking Pi-Sulfur	2.09 5.01 4.93
		(*R*)-N4-ATTAF-2	Tyr141 Phe237	Pi-Sulfur Pi-Sulfur	5.10 5.80
		(*S*)-N1-ATTAF-2	Phe242 Phe385 His382 Tyr127 Met512	Pi-Pi stacking Pi-Pi stacking Pi-Pi stacking Pi-Sulfur Pi-Sulfur	5.54 5.89 5.31 4.16 5.46
		(*S*)-N2-ATTAF-2	Tyr127 Ser383 His382	H-Bond H-Bond Pi-Pi stacking	2.86 2.32 5.36
		(*S*)-N4-ATTAF-2	Ser383 Phe385 Phe242 Tyr141 Met512	H-Bond Pi-Pi stacking Pi-Pi stacking Pi-Pi stacking Pi-Sulfur	2.61 5.08 5.59 5.45 4.83
3.	**3LD6**	(*R*)-N1-ATTAF-1	Thr135 Tyr131 Phe234 Tyr145 Phe139 Phe234	H-Bond Pi-Pi stacking Pi-Pi stacking Pi-Sulfur Pi-Sulfur Pi-Sulfur	3.16 4.79 5.11 4.80 5.51 5.60
		(*R*)-N2-ATTAF-1	Met378 Val130 Tyr131 Trp239	H-Bond H-Bond Pi-Pi stacking Donor-Donor (**Unfavorable**)	2.81 2.85 4.32 2.09
		(*R*)-N4-ATTAF-1	Tyr131 Phe139 Tyr145 Thr135 Phe139 Phe234	2 Pi-Pi stacking Pi-Pi stacking Pi-Sulfur Pi-Sulfur Pi-Sulfur Pi-Sulfur	4.18, 4.23 5.14 5.78 2.94 4.34 4.74
		(*S*)-N1-ATTAF-1	Thr135 Tyr145 Phe234 Tyr131	H-Bond H-Bond Pi-Pi stacking Pi-Sulfur	1.90 2.76 5.23 3.97
		(*S*)-N2-ATTAF-1	Tyr145 Tyr131 Phe139	2 H-Bond 2 Pi-Pi stacking Pi-Pi stacking	2.82, 3.54 4.13, 4.73 5.09
		(*S*)-N4-ATTAF-1	Thr135 Phe234 Phe234	H-Bond Pi-Pi stacking Pi-Sulfur	2.91 5.06 5.64
		(*R*)-N1-ATTAF-2	Tyr131 Thr135 Tyr145 Phe234	H-Bond H-Bond H-Bond Pi-Pi stacking	2.53 2.64 3.49 4.84
		(*R*)-N2-ATTAF-2	Ile379 Phe234	H-Bond Pi-Sulfur	2.25 5.89
		(*R*)-N4-ATTAF-2	Tyr131 Met381 Phe234	Pi-Pi stacking Pi-Sulfur Pi-Sulfur	4.27 4.94 5.99
		(*S*)-N1-ATTAF-2	Pro379 Met378	H-Bond Donor-Donor (**Unfavorable**)	2.29 2.51
		(*S*)-N2-ATTAF-2	Ala311 Ile379 Met381	Pi-Alkyl Pi-Alkyl Pi-Alkyl	4.34 4.45 4.30
		(*S*)-N4-ATTAF-2	Leu310 Ala311 Phe234	H-Bond H-Bond Pi-Pi stacking	2.68 2.99 4.21

## 2 Experimental design, materials and methods

### 2.1 Protein selection and preparation

The crystal structures of the selected proteins were retrieved from protein data bank. (PDB database, www.rcsb.org). Protein preparation was done by preprocessing the structures by removing water molecules, ions and cocrystallized ligands, polar hydrogens addition and assigning Gasteiger-Marsili partial charges, adjusting bonds and formal charges for metals, and removing unwanted chains. In order to rmsd validation, the co-crystallized ligand was re-docked. The target input files were converted to PDBQT format for AutoDock by using the AutoDockTools-1. 5. 4.

### 2.2 Ligand preparation and molecular docking

Ligands 3D structures were sketched by using ChemDraw Ultra 8.0 and energy minimized using PM3 force field. For all ligands, the nonpolar hydrogen atoms were merged and the Gasteiger charges were assigned. Then set number of torsion with detect root and choose torsion in Autodock program. Later, ligand input files were also saved as PDBQT format utilizing the AutoDock Tools. The minimized structures were docked on the prepared protein.

Discovery Studio Client 2016 and Molegro Molecular Viewer were used for further analysis.

## Declaration of Competing Interest

The authors declare that they have no known competing financial interests or personal relationships that could have appeared to influence the work reported in this paper.

## Appendix Supplementary materials

Image, application 1
