# Supplementary material for: Data on molecular docking of tautomers and enantiomers of ATTAF-1 and ATTAF-2 selectivty to the human/fungal lanosterol-14α-demethylase
Source: Data Brief. 2020 Jun 27;31:105942. doi: 10.1016/j.dib.2020.105942 (PMC7341365; doi:10.1016/j.dib.2020.105942)
Supplement: Supplementary file 1 [file mmc1.doc]

**Supplementary material**

**Data on molecular docking of tautomers and enantiomers of ATTAF-1 and ATTAF-2 selectivty to the human/fungal lanosterol-14α-demethylase**

Hamid Irannejad^a,b^, Saeed Emami^a,b^, Hassan Mirzaei^c^, Seyedeh Mahdieh Hashemi^a,b^*

*^a^Department of Medicinal Chemistry, Faculty of Pharmacy, Mazandaran University of Medical Sciences, Sari, Iran*

*^b^ Pharmaceutical Sciences Research Center, Mazandaran University of Medical Sciences, Sari, Iran*

*^c^Ischemic Disorders Research Center, Golestan University of Medical Sciences, Gorgan, Iran.*

*Corresponding author:

Seyedeh Mahdieh Hashemi, PhD

Department of Medicinal Chemistry and Pharmaceutical Sciences Research Center, Faculty of Pharmacy, Mazandaran University of Medical Sciences, Sari, Iran

Email: [hashemi325@gmail.com](mailto:hashemi325@gmail.com)

Tel: 0098 9112122579

Fax: 0098 1133543084

Postal code: 4847116547

https://orcid.org/0000-0002-8889-4146

**Content:**

- 2D binding mode of 8 standard triazole drugs, tautomers and enantiomers of ATTAF-1 and ATTAF-2 in *Candida albicans* CYP51 active site.
- 2D binding mode of 8 standard triazole drugs, tautomers and enantiomers of ATTAF-1 and ATTAF-2 in *Candida glabrata* CYP51 active site.
- 2D binding mode of 8 standard triazole drugs, tautomers and enantiomers of ATTAF-1 and ATTAF-2 in human CYP51 active site.

Abbreviations: CACYP51: *Candida albicans* CYP51, CGCYP51: *Candida glabrata* CYP51, hCYP51: human CYP51


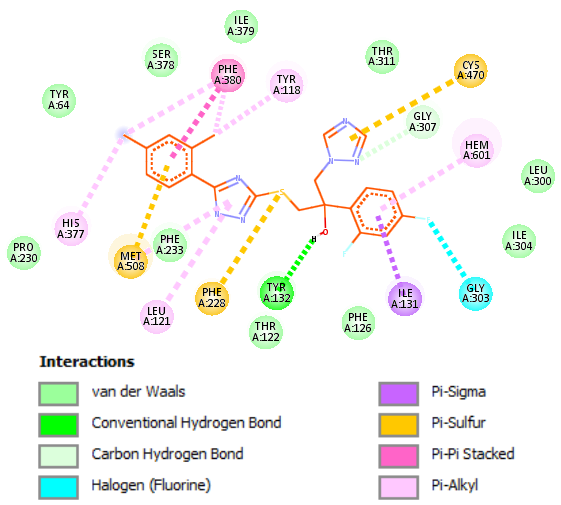

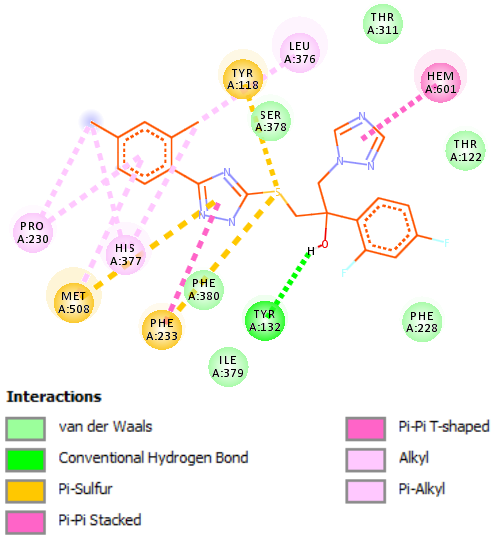


**(*R*)-N1-ATTAF-1/CACYP51 (*R*)-N2-ATTAF-1/CACYP51**


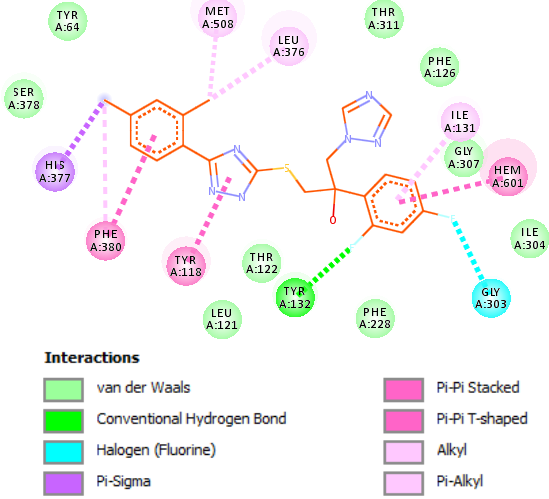

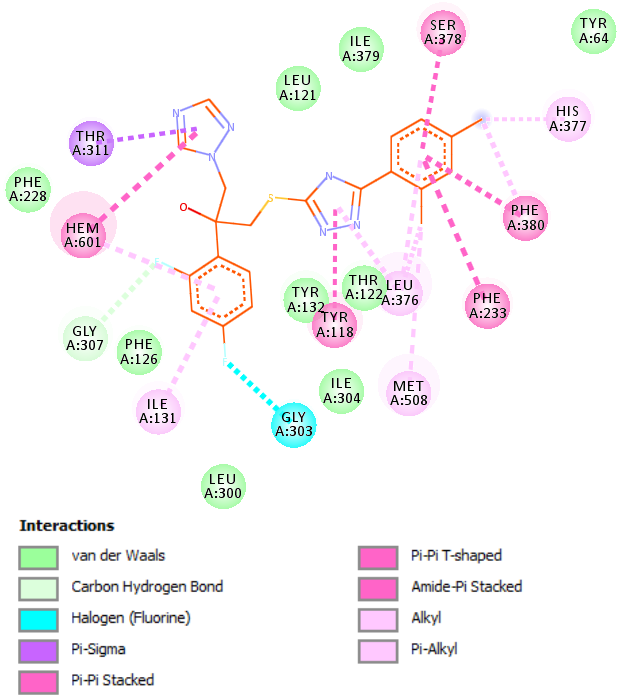


**(*R*)-N4-ATTAF-1/CACYP51** **(*S*)-N1-ATTAF-1/CACYP51**


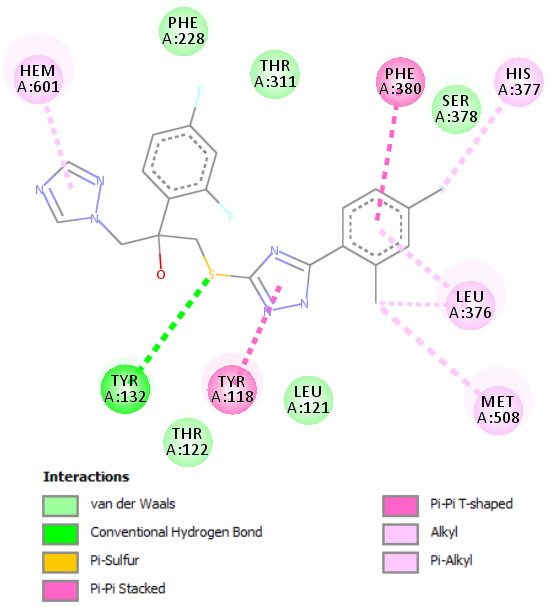

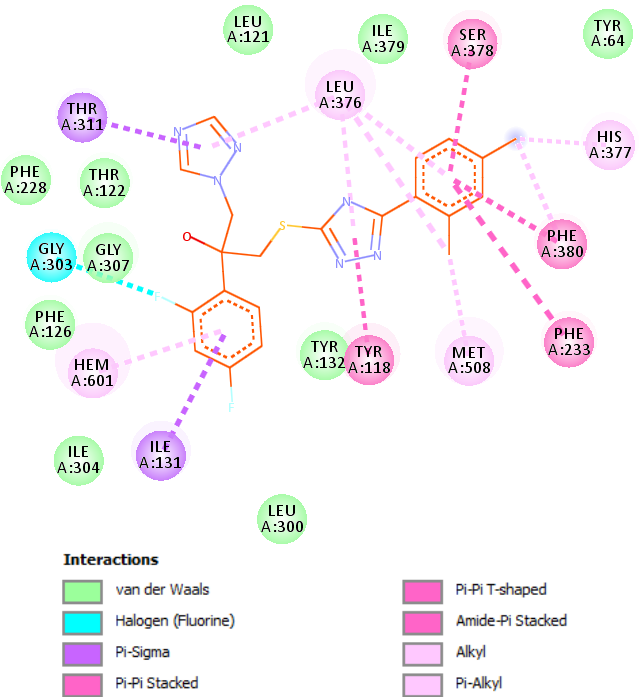


**(*S*)-N2-ATTAF-1/CACYP51**  **(*S*)-N4-ATTAF-1/CACYP51**


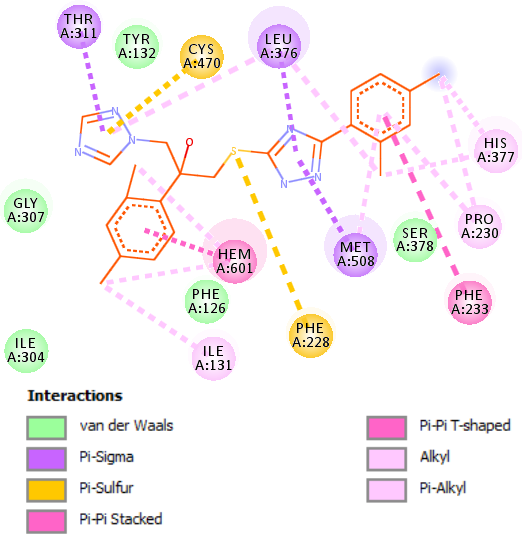

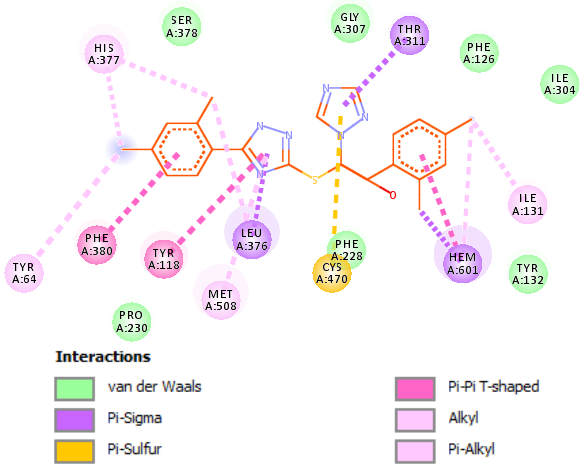


**(*R*)-N1-ATTAF-2/CACYP51 (*R*)-N2-ATTAF-2/CACYP51**


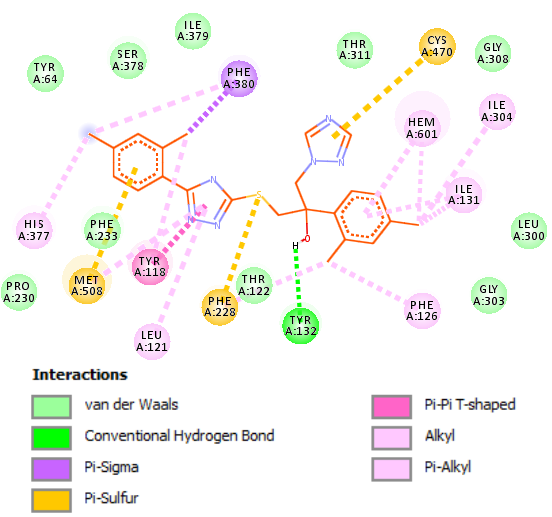

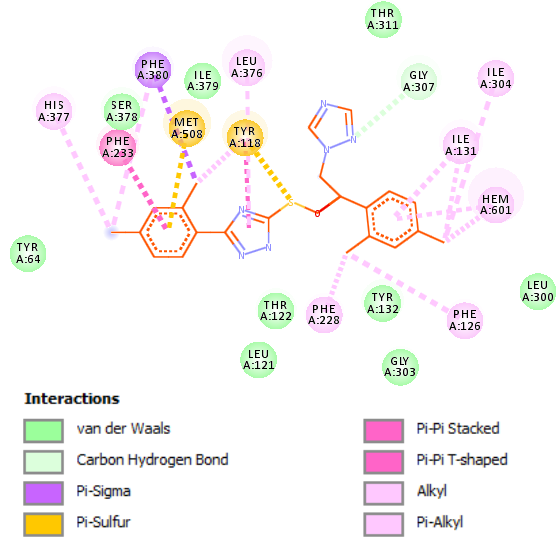


**(*R*)-N4-ATTAF-2/CACYP51 (*S*)-N1-ATTAF-2/CACYP51**


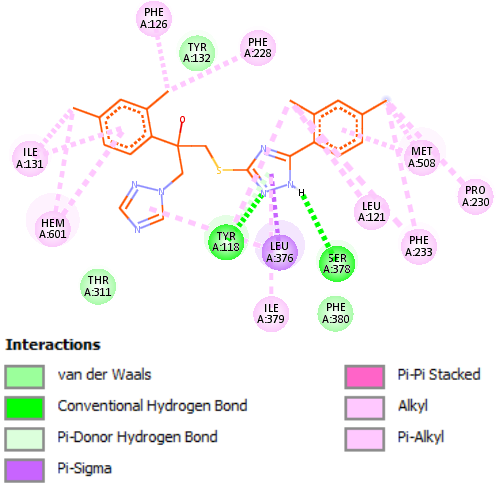

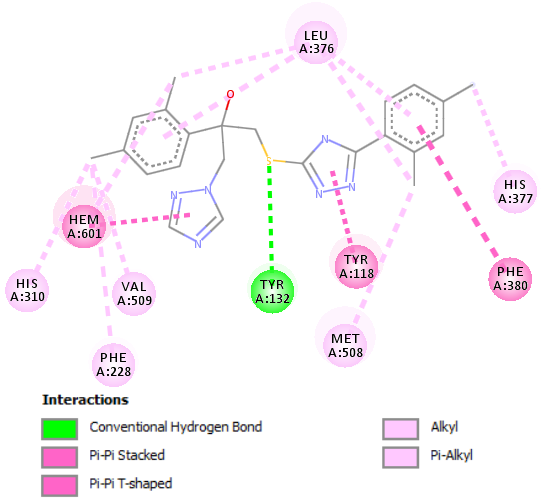


**(*S*)-N2-ATTAF-2/CACYP51 (*S*)-N4-ATTAF-2/CACYP51**

**Fluconazole/CACYP51**


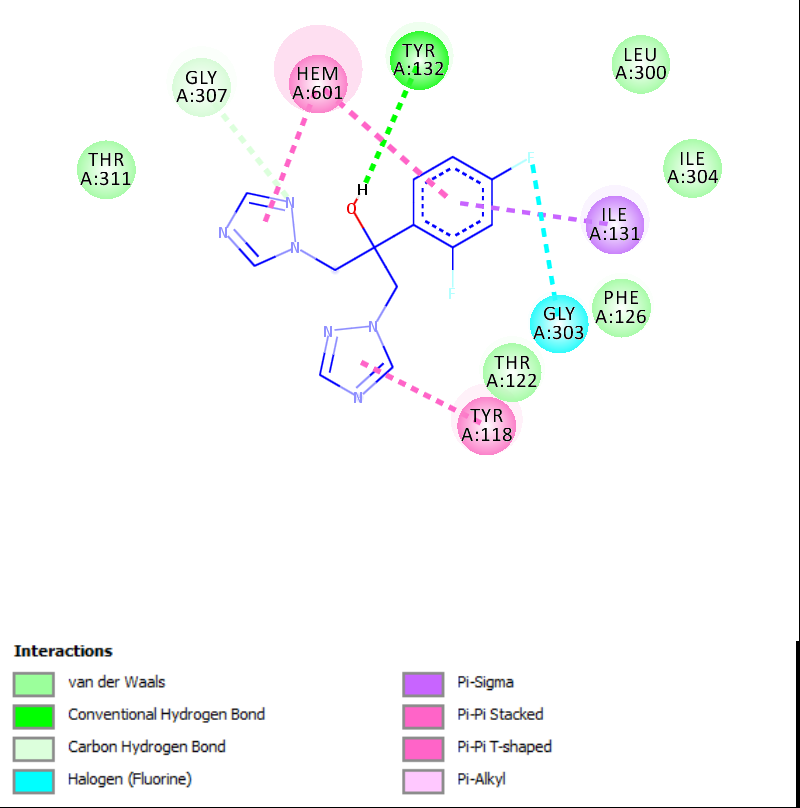


**Albaconazole/CACYP51**

**
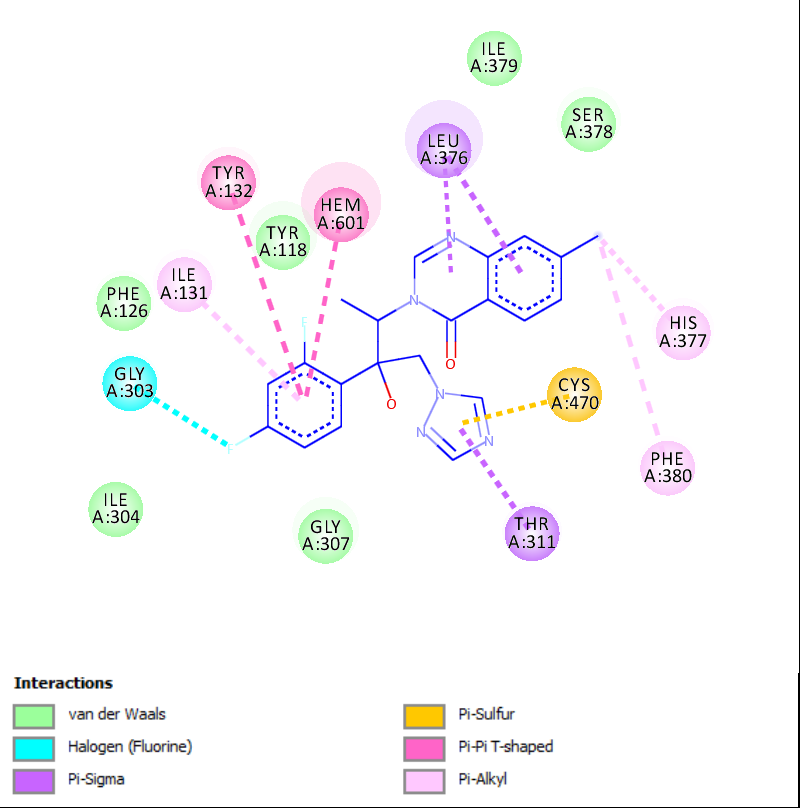
**

**Efinaconazole/CACYP51**

**
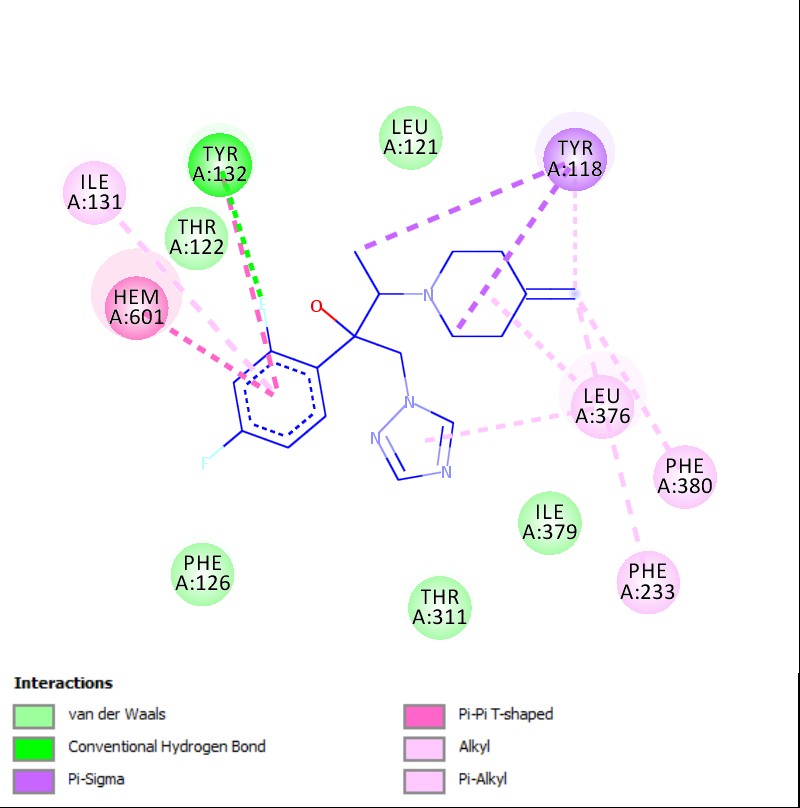
**

**Isavuconazole/CACYP51**

**
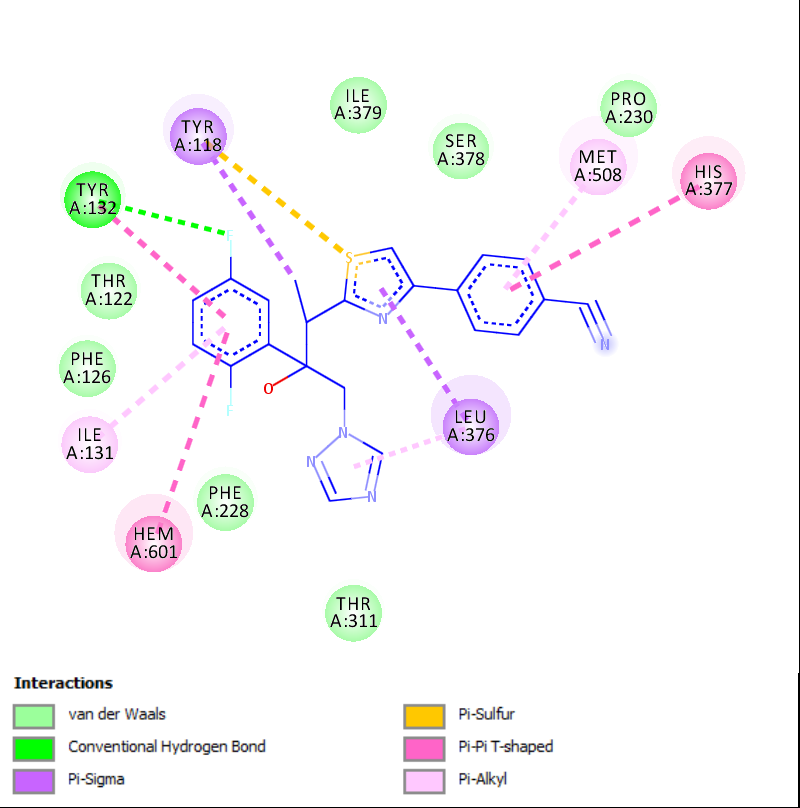
**

**Itraconazole/CACYP51**

**
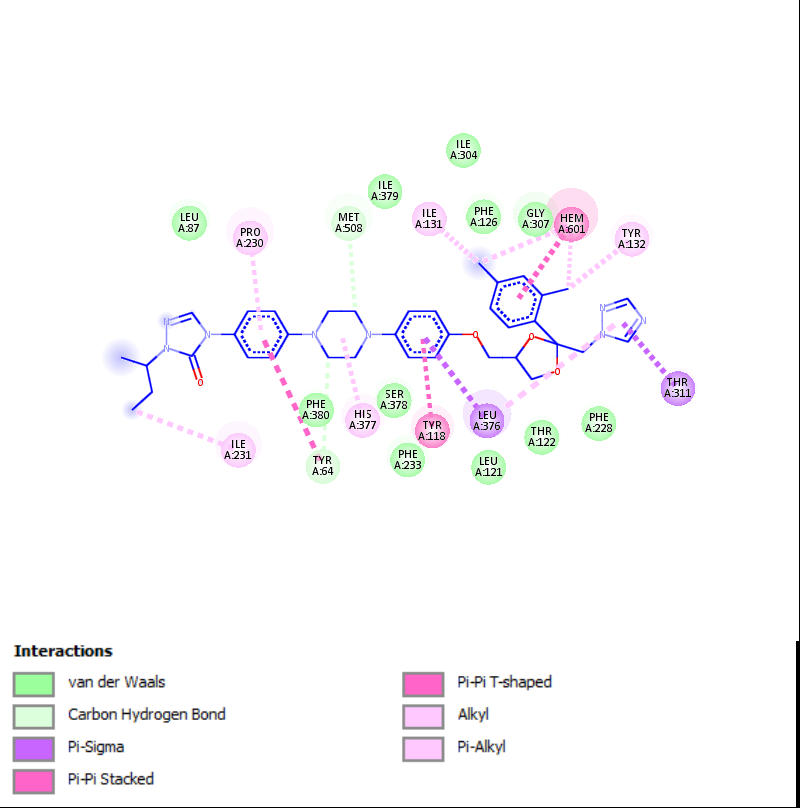
**

**Posaconazole/CACYP51**

**
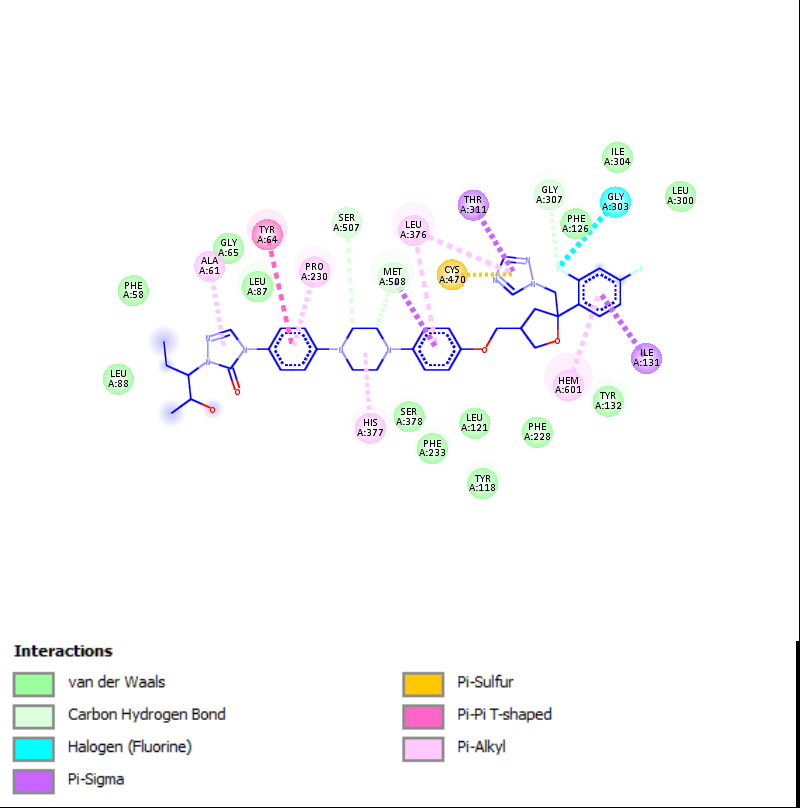
**

**Ravuconazole/CACYP51**

**
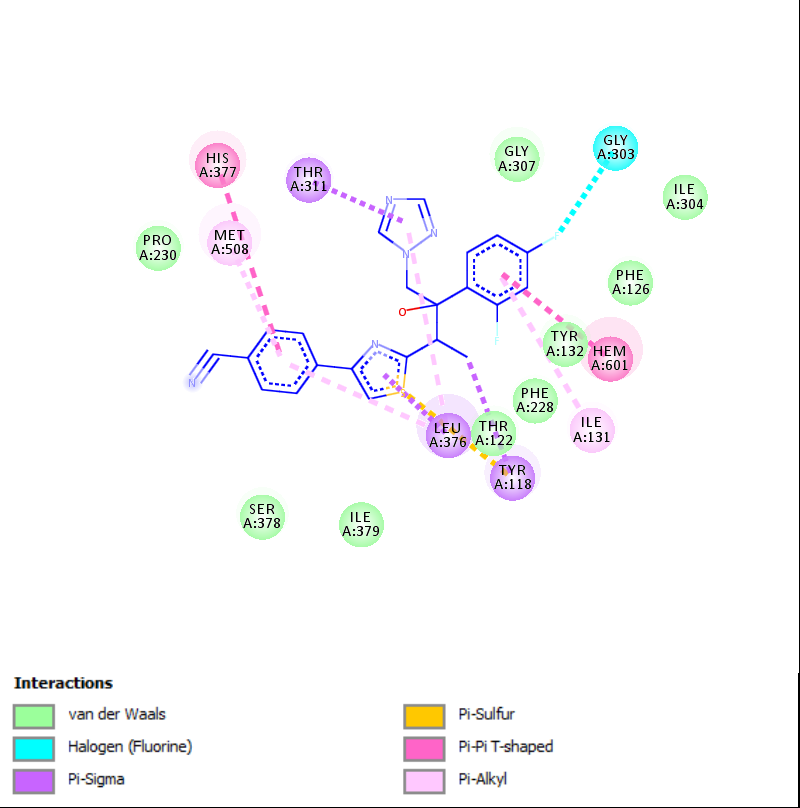
**

**Voriconazole/CACYP51**

**
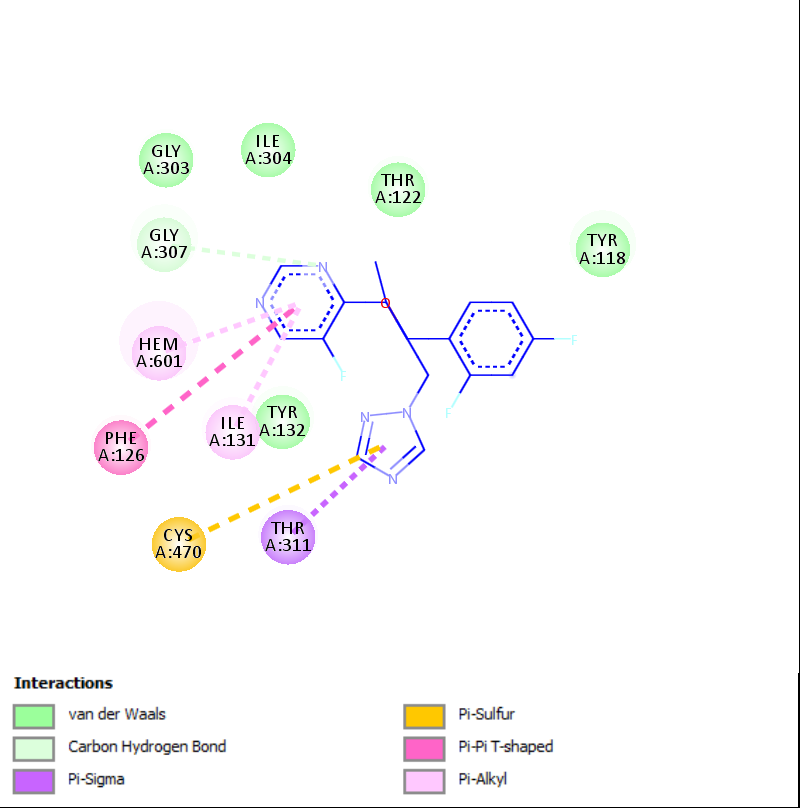
**

**Fig. 1.** Ligand interaction map of the predicted binding mode of 8 standard triazole drugs, tautomers and enantiomers of ATTAF-1 and ATTAF-2 in the active site *Candida albicans* CYP51**.**


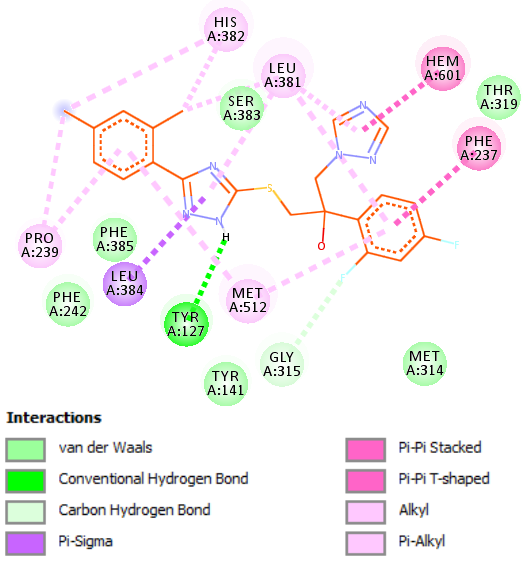

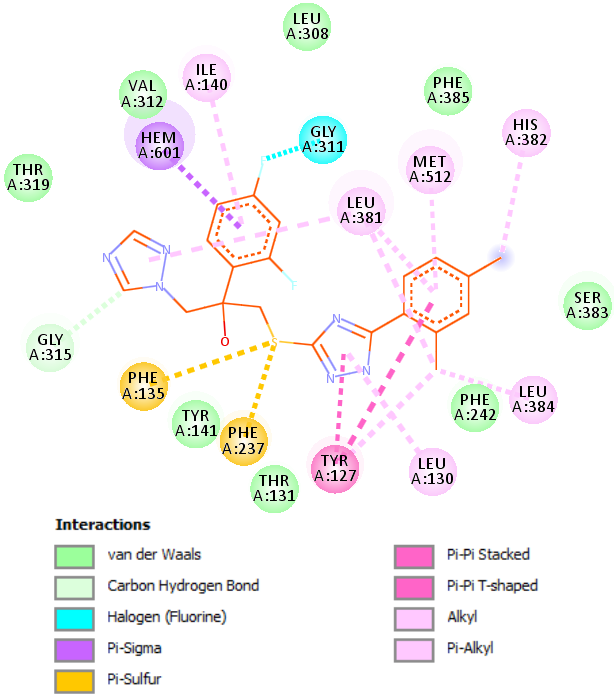


**(*R*)-N1-ATTAF-1/CGCYP51 (*R*)-N2-ATTAF-1/CGCYP51**


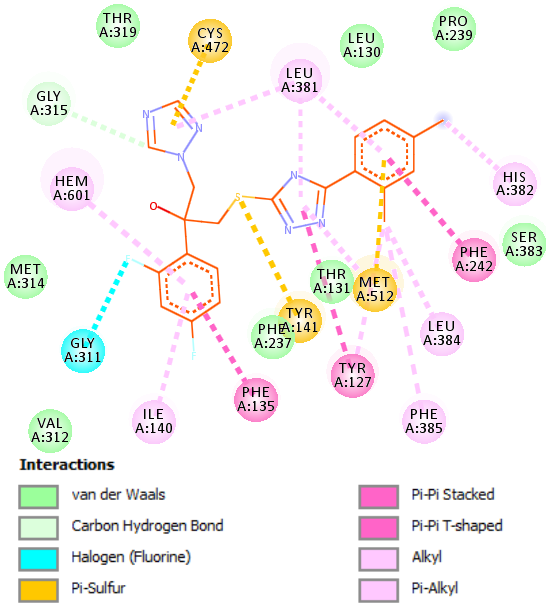

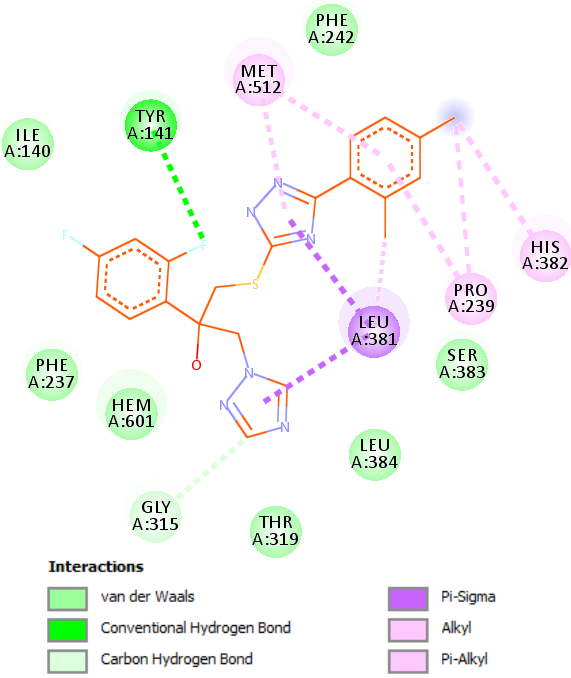


**(*R*)-N4-ATTAF-1/CGCYP51 (*S*)-N1-ATTAF-1/CGCYP51**


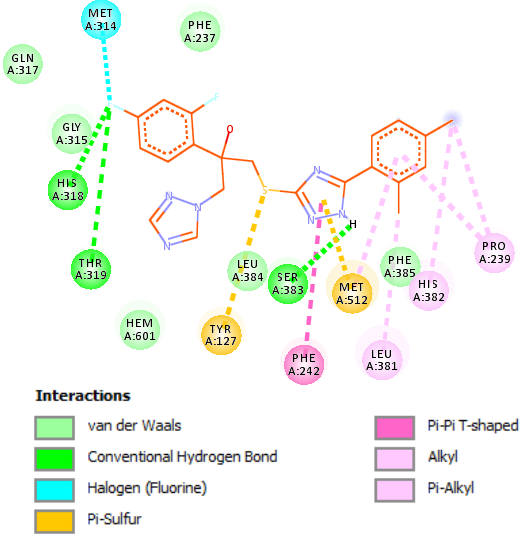

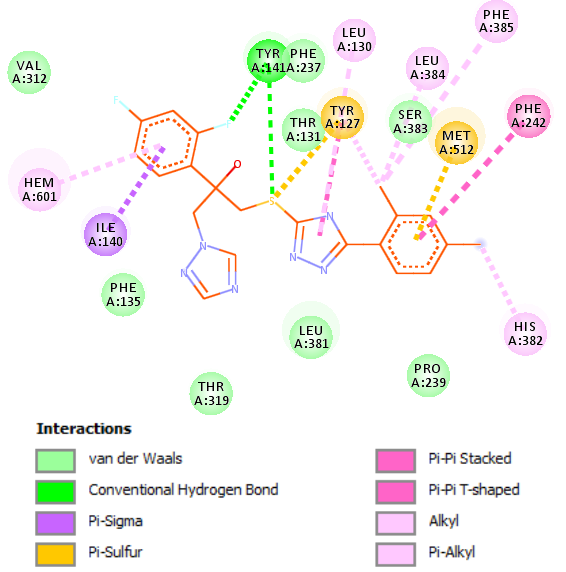


**(*S*)-N2-ATTAF-1/CGCYP51 (*S*)-N4-ATTAF-1/CGCYP51**

**
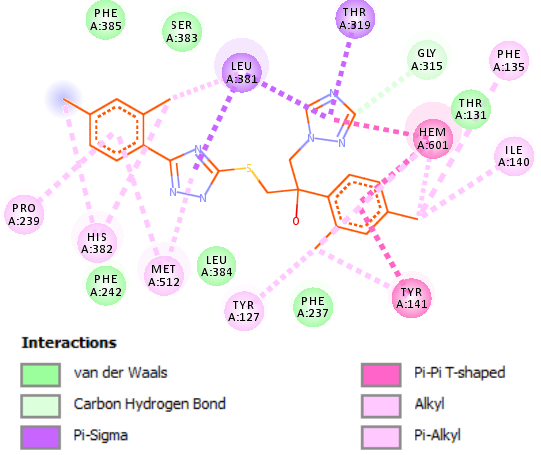
**
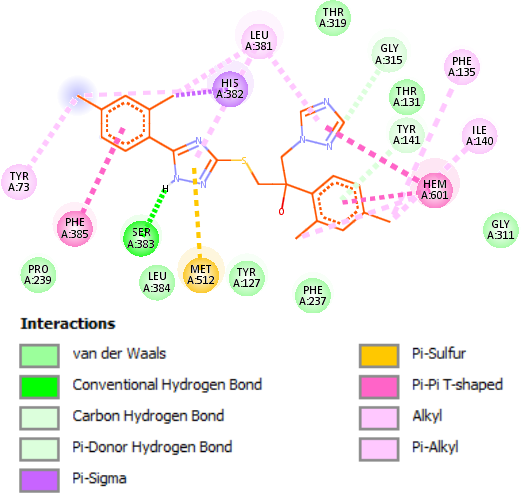


**(*R*)-N1-ATTAF-2/CGCYP51 (*R*)-N2-ATTAF-2/CGCYP51**


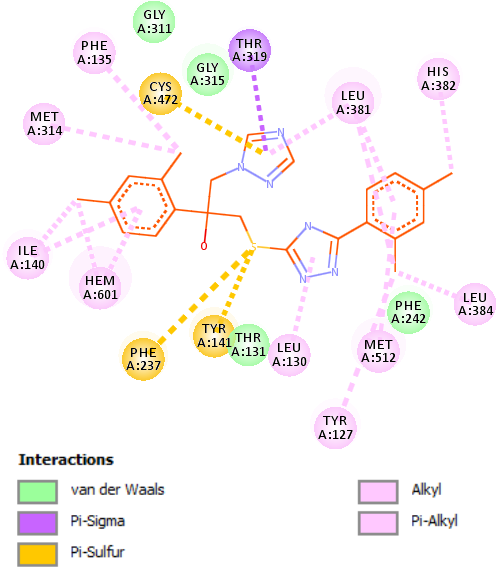

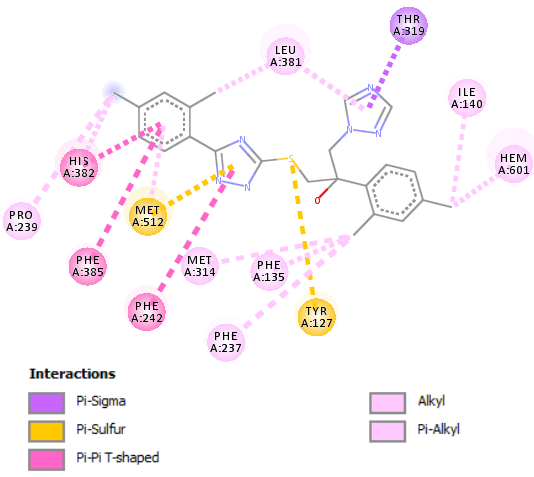


**(*R*)-N4-ATTAF-2/CGCYP51 (*S*)-N1-ATTAF-2/CGCYP51**


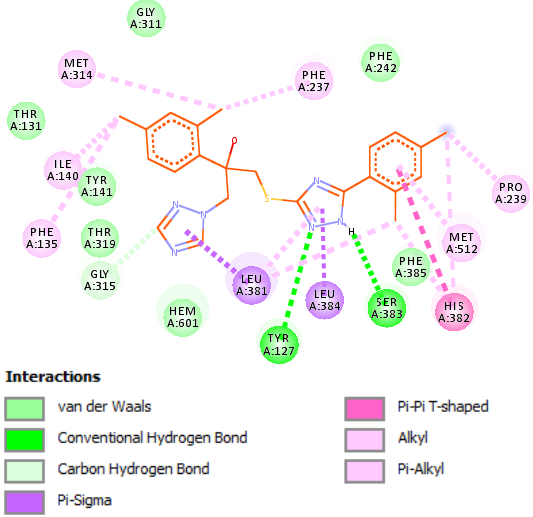

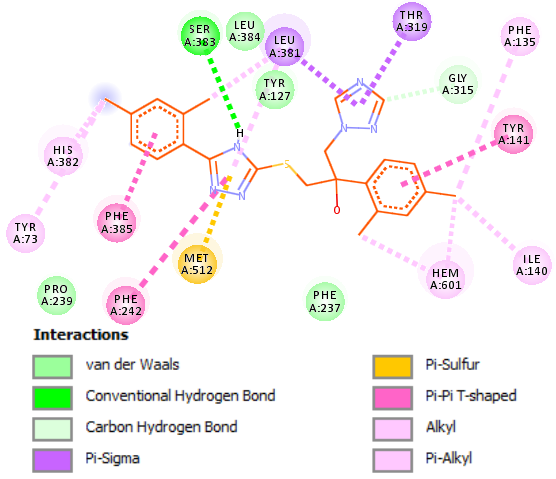


**(*S*)-N2-ATTAF-2/CGCYP51 (*S*)-N4-ATTAF-2/CGCYP51**

**Fluconazole/CGCYP51**


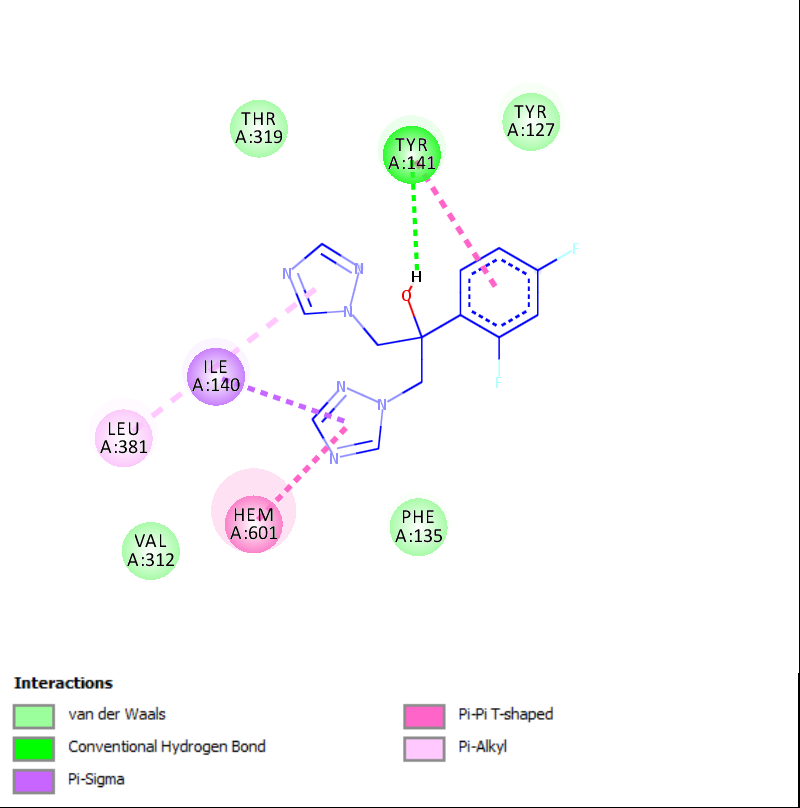


**Albaconazole/CGCYP51**

**
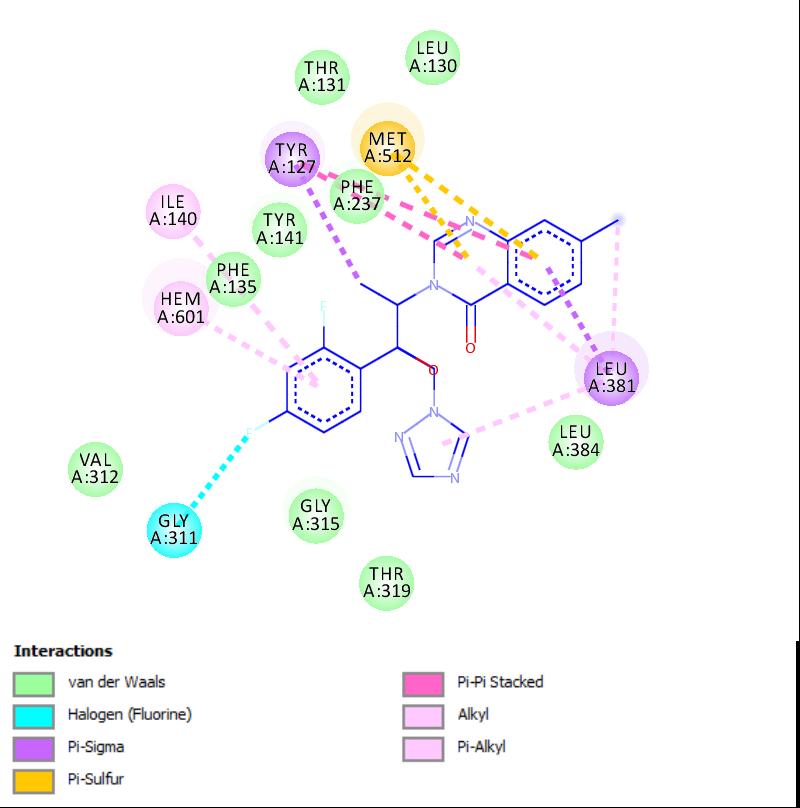
**

**Posaconazole/CGCYP51**

**
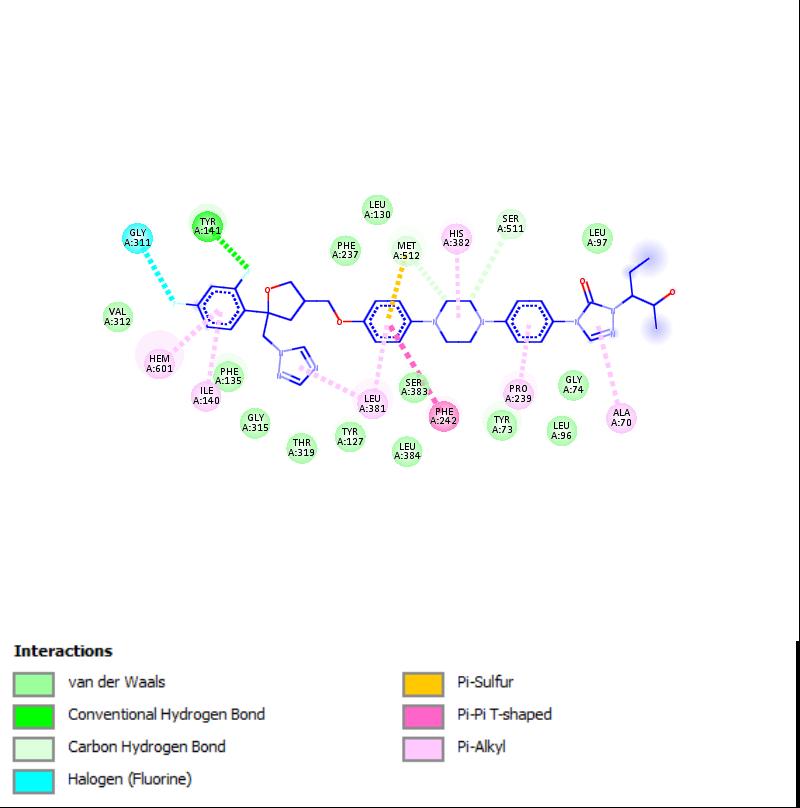
**

**Voriconazole/CGCYP51**

**
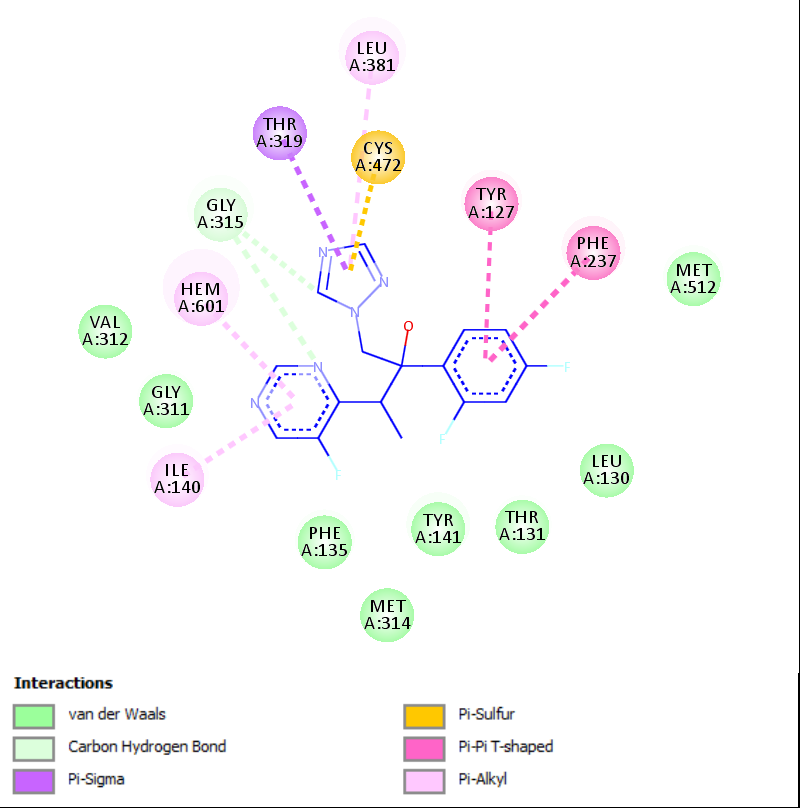
**

**Ravuconazole/CGCYP51**

**
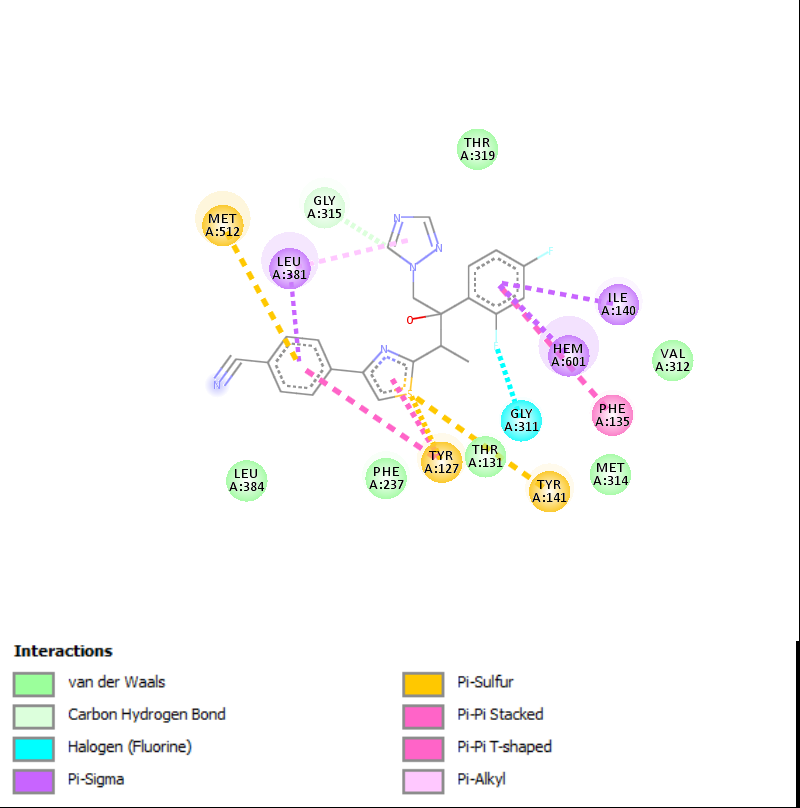
**

**Isavuconazole/CGCYP51**

**
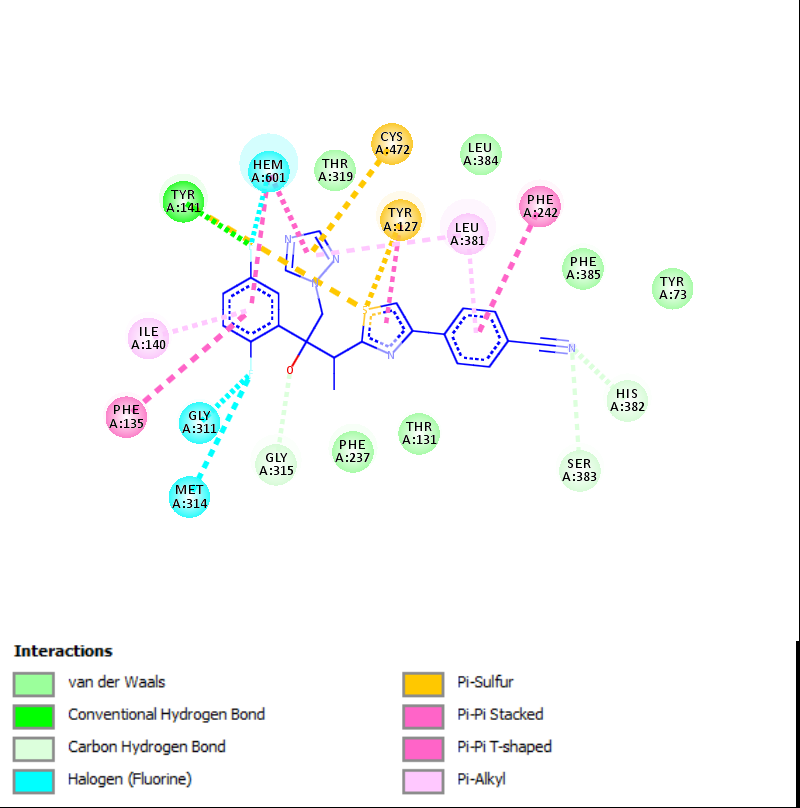
**

**Fig. 2.** Ligand interaction map of the predicted binding mode of 8 standard triazole drugs, tautomers and enantiomers of ATTAF-1 and ATTAF-2 in the active site *Candida glabrata* CYP51**.**


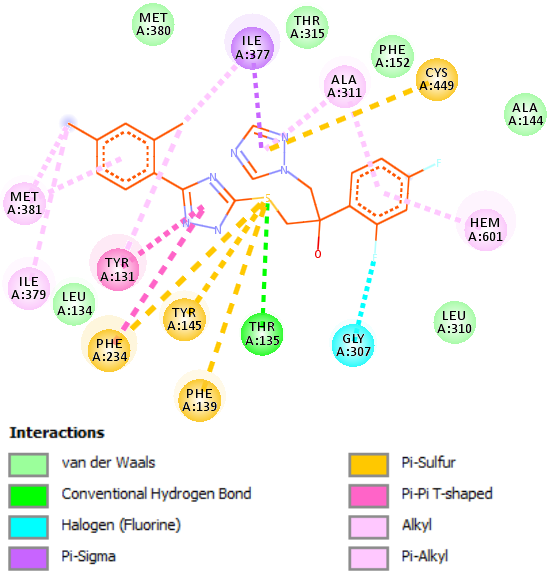

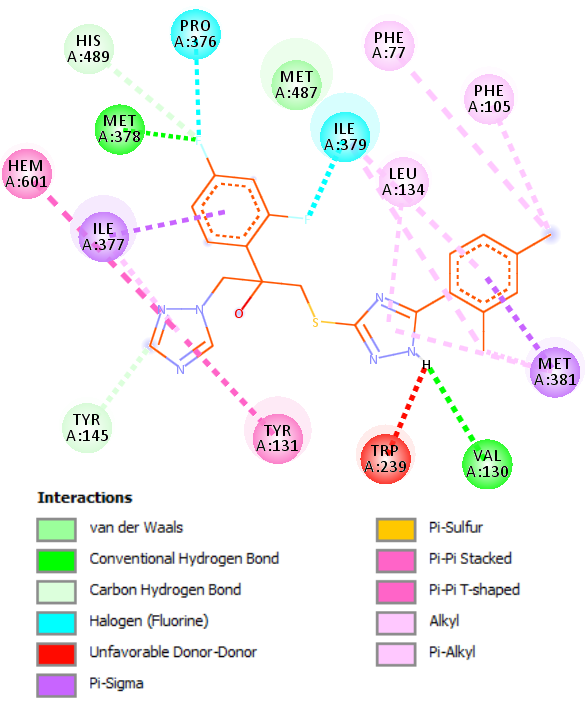


**(*R*)-N1-ATTAF-1/hCYP51 (*R*)-N2-ATTAF-1/hCYP51**


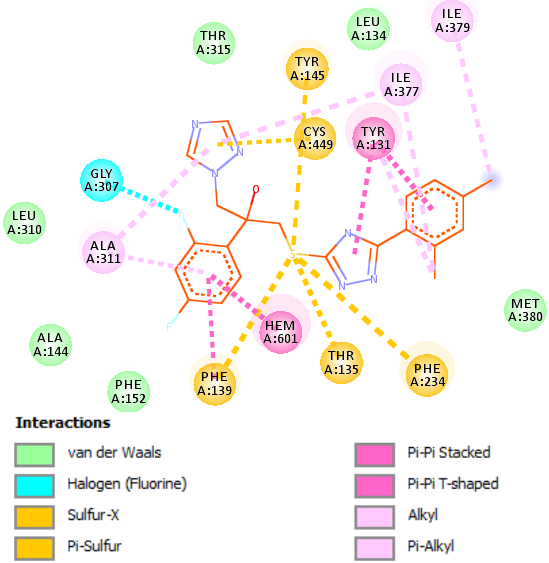

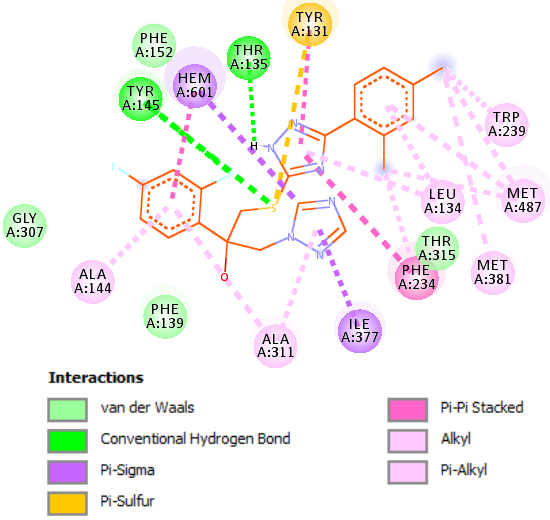


**(*R*)-N4-ATTAF-1/hCYP51 (*S*)-N1-ATTAF-1/hCYP51**


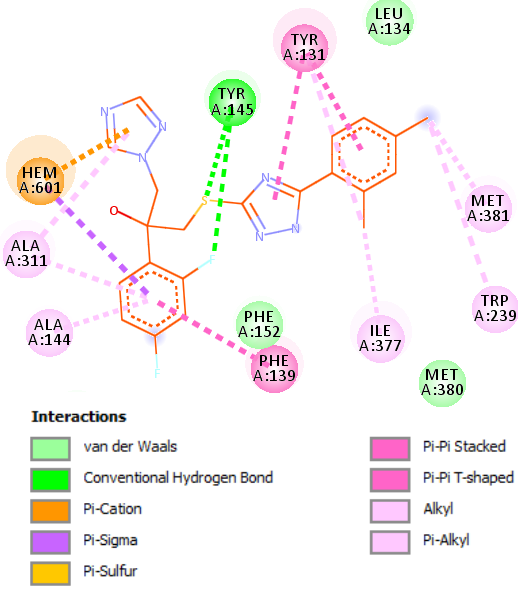

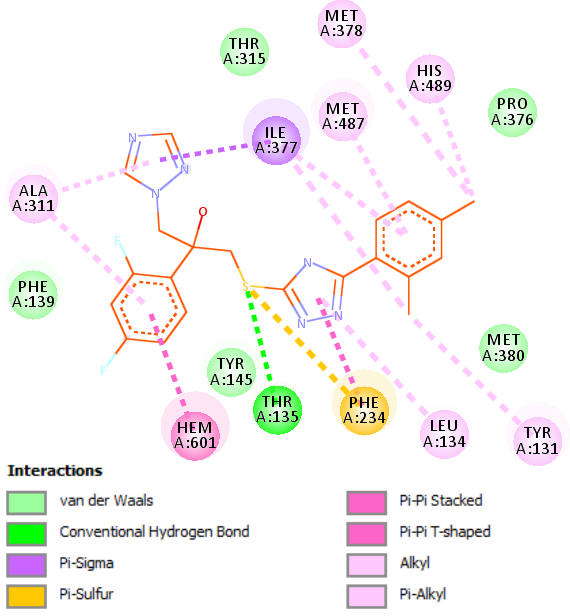


**(*S*)-N2-ATTAF-1/hCYP51** **(*S*)-N4-ATTAF-1/hCYP51**


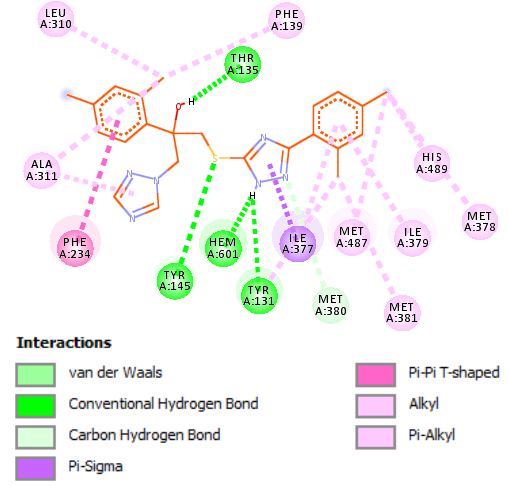

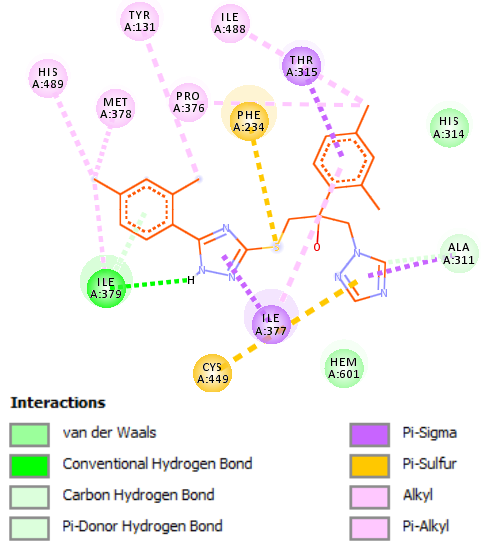


**(*R*)-N1-ATTAF-2/hCYP51 (*R*)-N2-ATTAF-2/hCYP51**

**
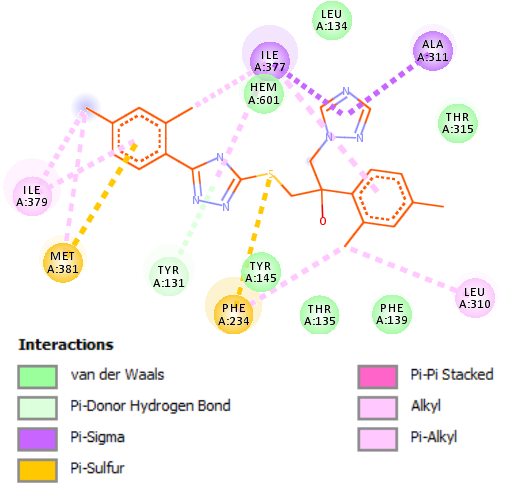
**
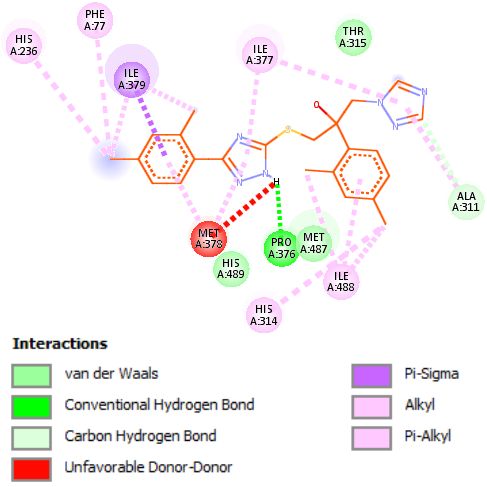


**(*R*)-N4-ATTAF-2/hCYP51 (*S*)-N1-ATTAF-2/hCYP51**


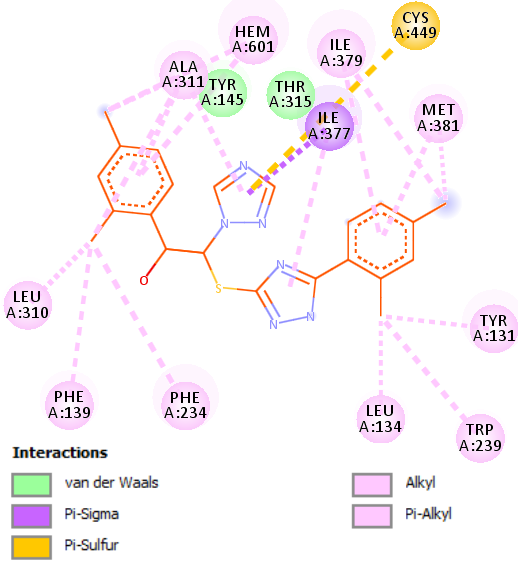
**
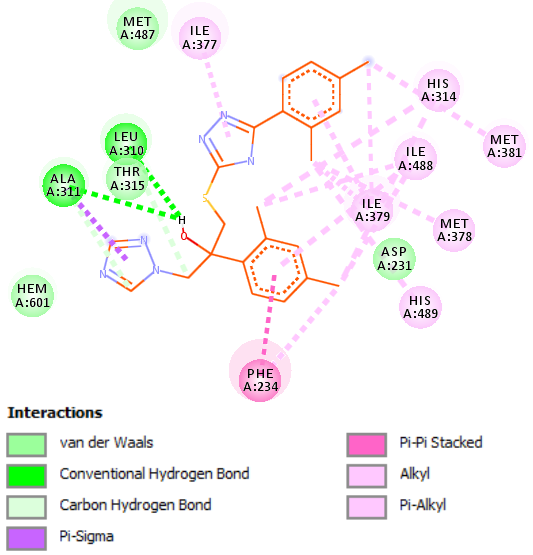
**

**(*S*)-N2-ATTAF-2/hCYP51 (*S*)-N4-ATTAF-2/hCYP51**

**Fluconazole/hCYP51**

**
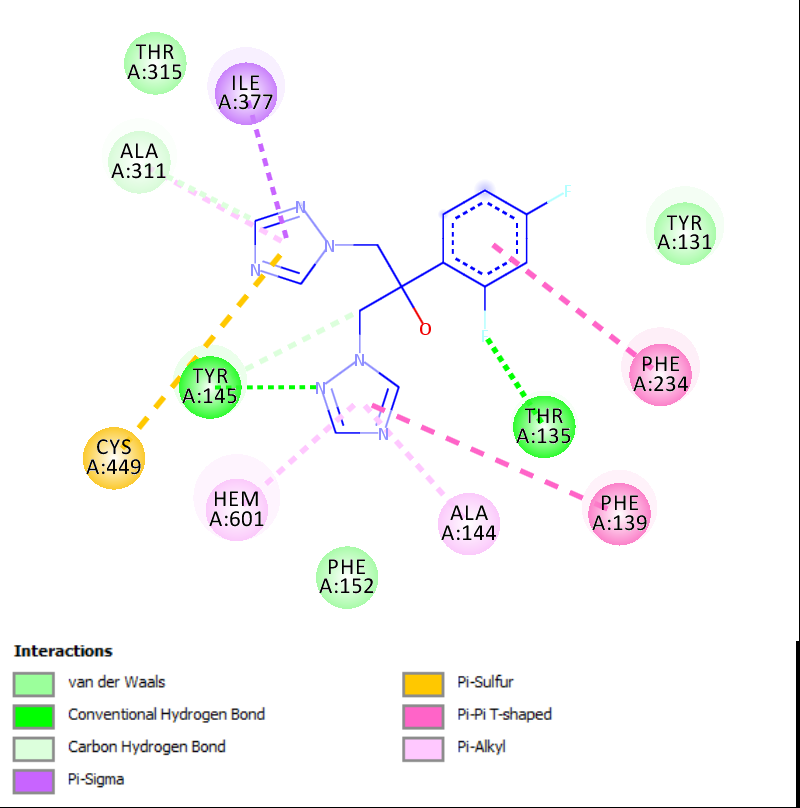
**

**Ravuconazole/hCYP51**

**
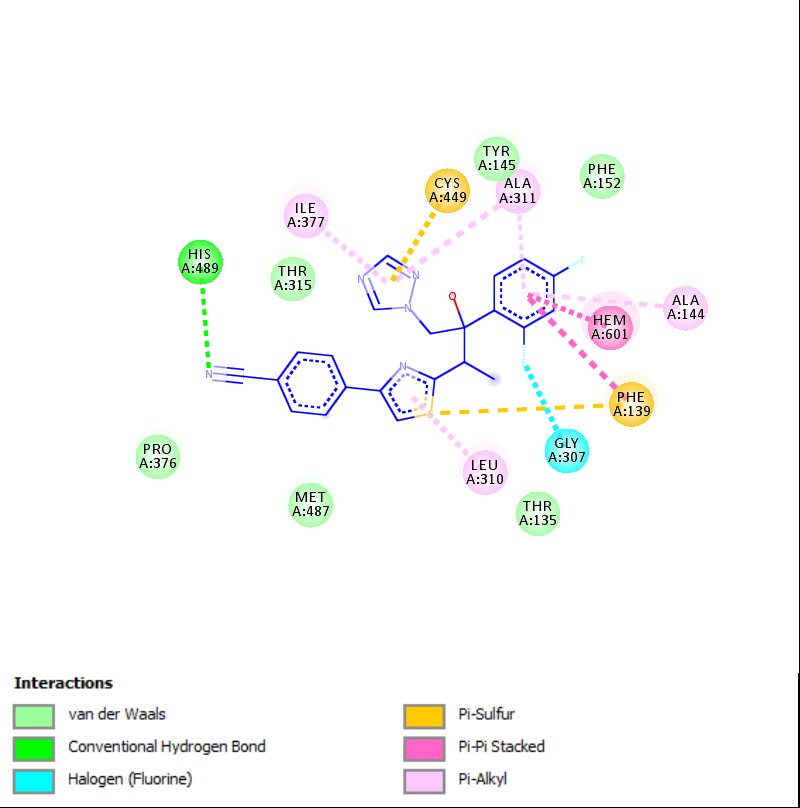
**

**Voriconazole/hCYP51**

**
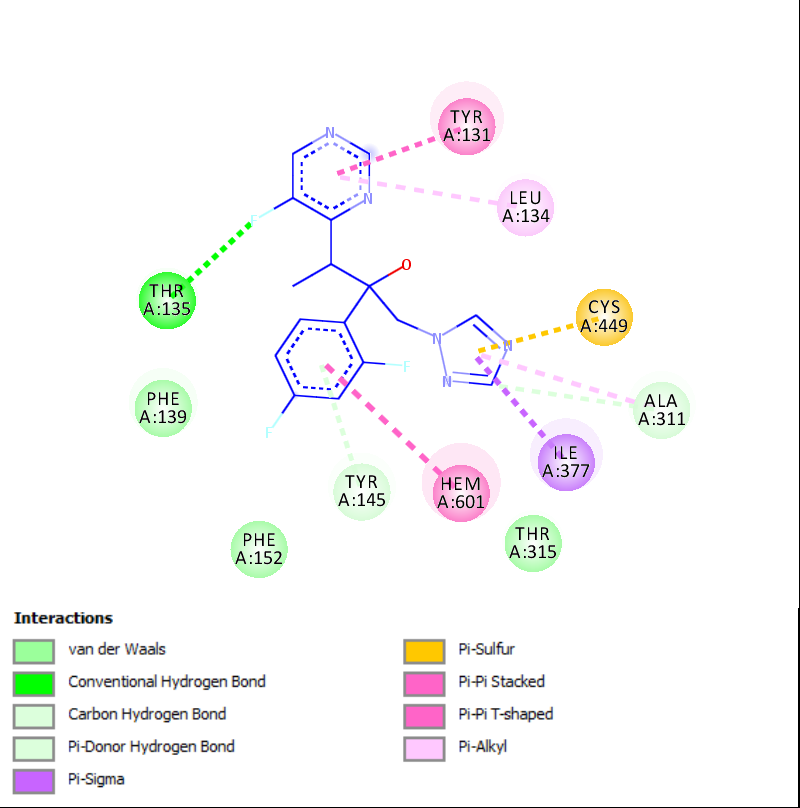
**

**Posaconazole/hCYP51**

**
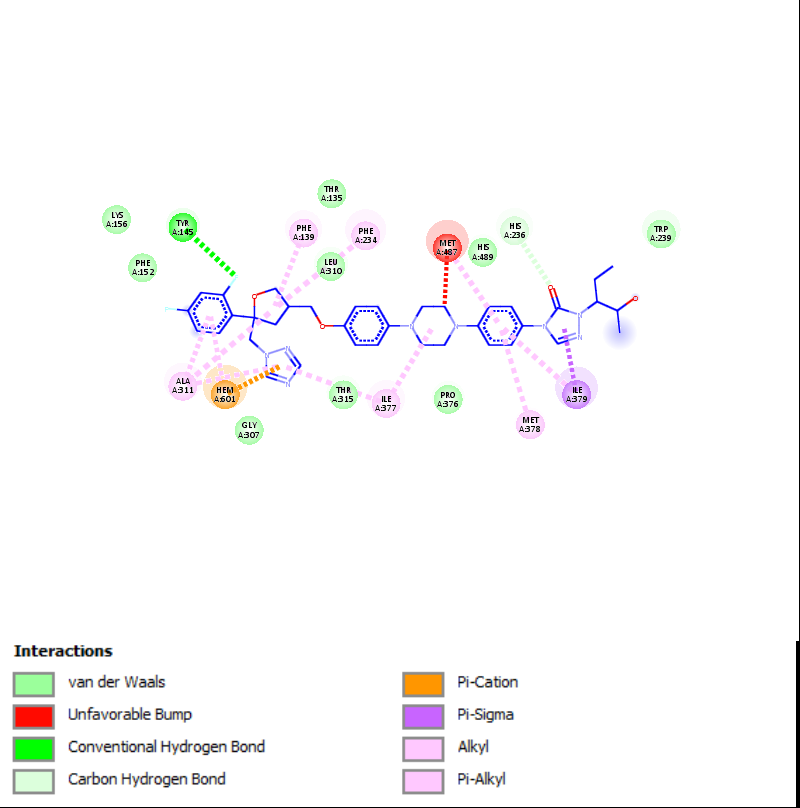
**

**Efinaconazole/hCYP51**

**
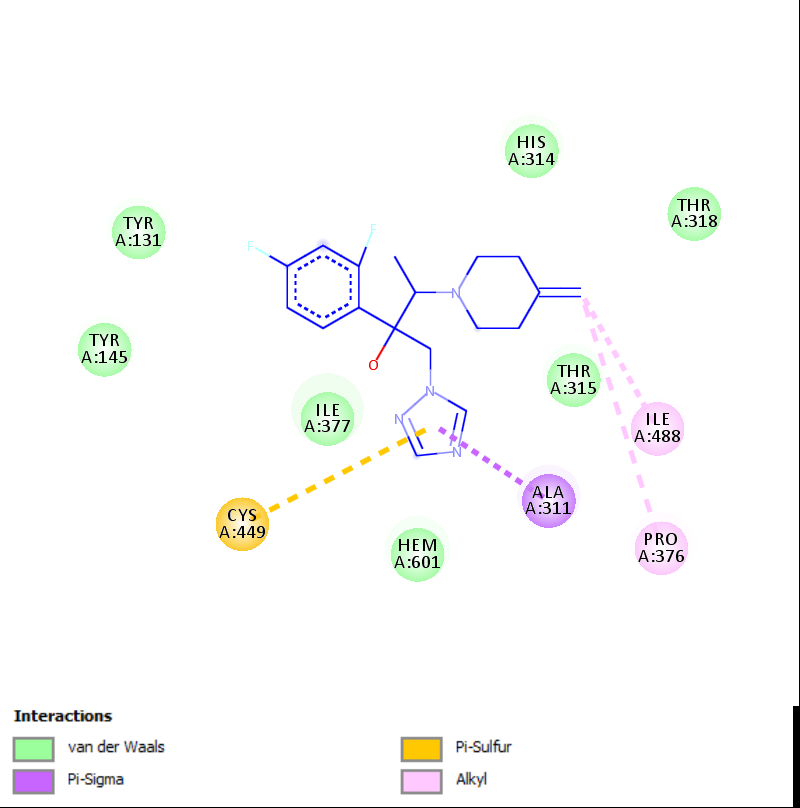
**

**Albaconazole/hCYP51**

**
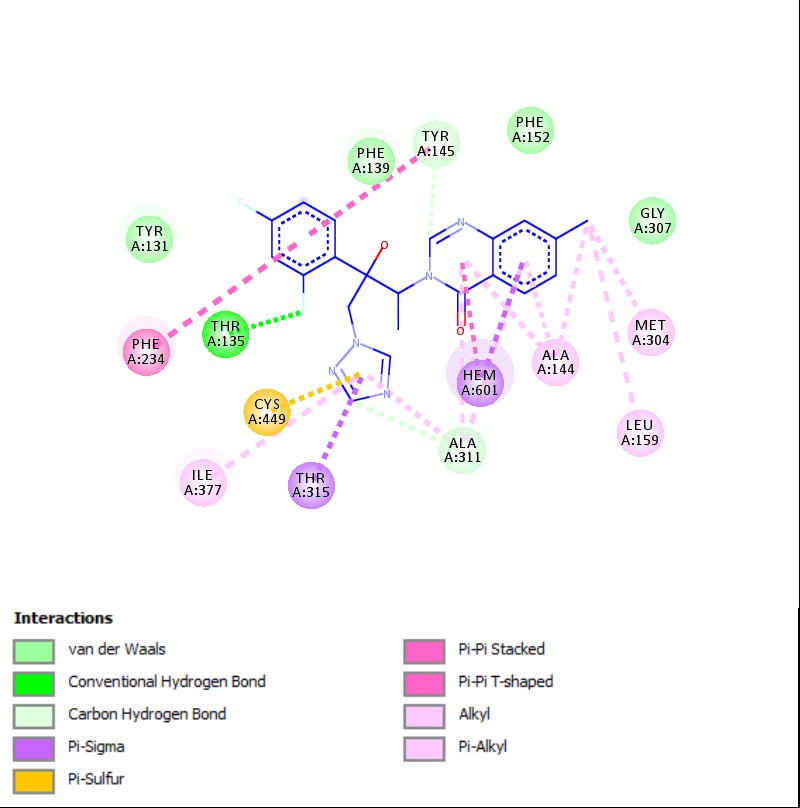
**

**Itraconazole/hCYP51**

**
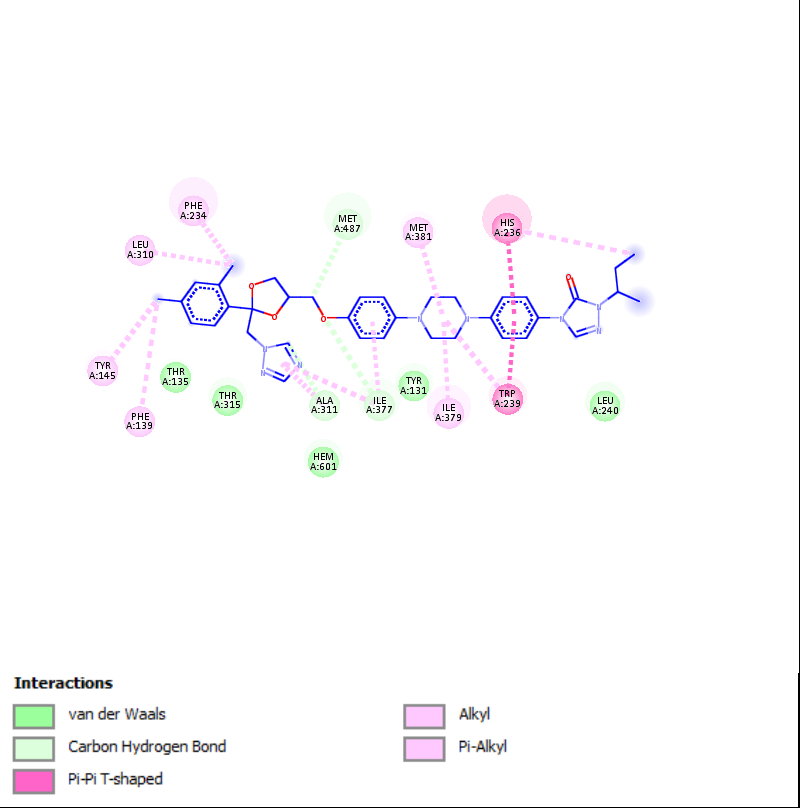
**

**Isavuconazole/hCYP51**

**
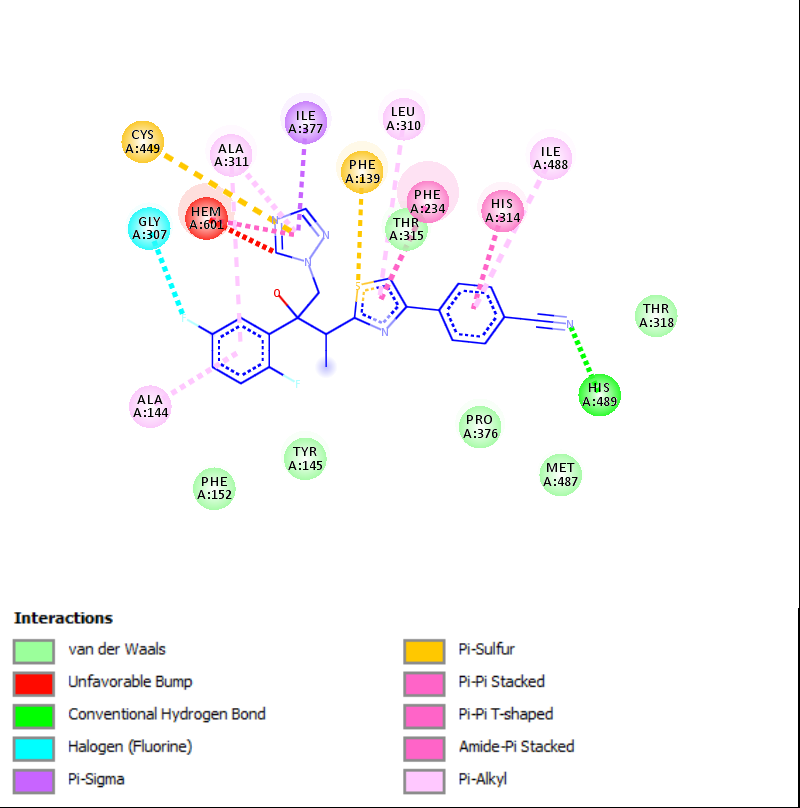
**

**Fig. 3.** Ligand interaction map of the predicted binding mode of 8 standard triazole drugs, tautomers and enantiomers of ATTAF-1 and ATTAF-2 in the active site human CYP51**.**
